# Capturing cell type-specific chromatin compartment patterns by applying topic modeling to single-cell Hi-C data

**DOI:** 10.1371/journal.pcbi.1008173

**Published:** 2020-09-18

**Authors:** Hyeon-Jin Kim, Galip Gürkan Yardımcı, Giancarlo Bonora, Vijay Ramani, Jie Liu, Ruolan Qiu, Choli Lee, Jennifer Hesson, Carol B. Ware, Jay Shendure, Zhijun Duan, William Stafford Noble

**Affiliations:** 1 Department of Genome Sciences, University of Washington, Seattle, Washington, United States of America; 2 Department of Biochemistry and Biophysics, University of California San Francisco, San Francisco, California, United States of America; 3 Department of Comparative Medicine, University of Washington, Seattle, Washington, United States of America; 4 Division of Hematology, Department of Medicine, University of Washington, Seattle, Washington, United States of America; 5 Institute for Stem Cell and Regenerative Medicine, University of Washington, Seattle, Washington, United States of America; 6 Paul G. Allen School of Computer Science & Engineering, University of Washington, Seattle, Washington, United States of America; University of Ottawa, CANADA

## Abstract

Single-cell Hi-C (scHi-C) interrogates genome-wide chromatin interaction in individual cells, allowing us to gain insights into 3D genome organization. However, the extremely sparse nature of scHi-C data poses a significant barrier to analysis, limiting our ability to tease out hidden biological information. In this work, we approach this problem by applying topic modeling to scHi-C data. Topic modeling is well-suited for discovering latent topics in a collection of discrete data. For our analysis, we generate nine different single-cell combinatorial indexed Hi-C (sci-Hi-C) libraries from five human cell lines (GM12878, H1Esc, HFF, IMR90, and HAP1), consisting over 19,000 cells. We demonstrate that topic modeling is able to successfully capture cell type differences from sci-Hi-C data in the form of “chromatin topics.” We further show enrichment of particular compartment structures associated with locus pairs in these topics.

## Author summary

The genomes of higher organisms are intricately folded and organized in a dynamic manner that has strong implications for many biological processes. Each chromosome undergoes dramatic changes to their three dimensional conformation during the cell cycle, whereas the positioning of chromosomes within the nucleus plays an important role in controlling the activation of specific genes. Recently, it has become possible to investigate the 3D conformations of the genomes of individual cells using a high throughput sequencing assay called single cell Hi-C (scHi-C). However, data from these assays are sparse and noisy, making analysis and interpretation of scHi-C data challenging. In this work, we generated a scHi-C dataset of over 19,000 cells from five human cell lines and applied a natural language processing method called topic modeling to discover cell type-specific “chromatin” topics. We show that these topics can be used to distinguish between cells at different stages of the cell cycle and cells from different tissues based on the 3D conformation of their genomes, despite the sparsity of the data. We further show that the 3D conformations of single cells are linked to the expression of cell type-specific genes and to cell cycle-associated conformational patterns.

## Introduction

Single-cell chromosome conformation capture methods, such as single-cell Hi-C (scHi-C), [1, 2, 3, 4, 5, 6] enable quantitative assessment of 3D conformation of chromosomes in individual cells. The resulting data can be used to detect cell-to-cell variations in genome-wide chromatin interactions and has the potential to interrogate chromosome structural heterogeneity in different cell types and states, thereby extracting important biological features of chromatin organization that are otherwise hidden in bulk Hi-C data. However, scHi-C measures only a very small fraction of extant chromosomal contacts in each individual cell and the resulting sparse nature of scHi-C data presents a major computational barrier to the analysis. The challenge becomes more acute when the number of cells is large. Standard dimensionality reduction techniques, such as Principal Component Analysis (PCA) [7], t-Distributed Stochastic Neighbor Embedding (t-SNE) [8], and Uniform Manifold Approximation and Projection (UMAP) [9], fail to retain the unique structure of high-dimensional scHi-C data.

Currently, a limited number of methods have been developed for the analysis of scHi-C data. We previously developed a similarity-based embedding method called HiCRep/MDS to project scHi-C data into low dimensional space and arrange cells according to their cell-cycle phases [10]. This approach leverages a similarity measure, stratum adjusted correlation coefficient, that was developed for comparing bulk Hi-C matrices [11], combined with multidimensional scaling (MDS) to preserve distances between individual scHi-C contact maps. However, HiCRep/MDS performs smoothing and pairwise comparison of the individual intra-chromosomal contact matrices, which makes the method computationally costly. Furthermore, although HiCRep/MDS can very accurately recover cell cycle information from several Hi-C data sets, the method frequently fails to capture chromatin structural differences between cell types [12].

More recently, Zhou *et al*. applied linear convolutions and random walks to scHi-C matrices to cluster cells according to their cell types [12]. This method uses imputation to reduce the sparsity of scHi-C matrices and identify chromatin structures that are similar to topologically associated domains within single cells. Despite the benefits this method offers, it lacks the ability to capture a set of genomic interactions that distinguish between cell types and to allow us to biologically interpret which features of chromatin organization drove the clustering.

To overcome these limitations, we turned our attention to topic modeling. Topic modeling is mainly used in natural language processing to tackle challenging problems in text mining such as discovering hidden structures (or “topics”) in large collections of sparse, discrete data. One widely used approach to topic modeling is latent Dirichlet allocation (LDA), an unsupervised generative probabilistic model for a collection of documents [13]. In this setting, each document is represented as a vector of word frequencies. The LDA models this vector as a mixture of topics, where each topic is represented as a probabilistic distribution over words. In the end, LDA generates two matrices, one that describes the relationship between topics and documents and the other describing the relationship between topics and words [14]. In the first matrix, topics with the highest contributions are those that best characterize a document. In the second matrix, words with the highest contributions in a topic provide a sense of what the topic might represent.

Recently, González-Blas *et al*. applied LDA topic modeling to single-cell epigenomic data to simultaneously discover cell types and latent topics that pertain to topic-specific regulatory programs [15]. This approach applied topic modeling to single-cell ATAC-seq data to predict cell type-specific gene regulatory networks in the human brain.

Motivated by this work, we hypothesized that LDA could be employed to extract biologically meaningful structure from single-cell Hi-C data. In our setting, each cell corresponds to a document, and each observed single cell Hi-C interaction between a pair of loci corresponds to a word. We call these interactions locus pairs. We aimed to uncover features of chromatin structures—chromatin topics—that are characteristic of particular cell types. In our work, we generate single-cell Hi-C data from five human cell lines (GM12878, H1Esc, HFF, IMR90, and HAP1) using single-cell combinatorial indexed Hi-C (sci-Hi-C) [3] and apply LDA topic modeling to the *in silico* mixed sci-Hi-C data. We show that LDA is able to demix the cell populations by cell types and discover cell type-specific topics of locus pairs. We demonstrate how the LDA identifies locus pairs that characterize each topic, and we show that the topics carry information about cell type-specific locus pair compartment structure.

## Materials and methods

### Datasets

Nine human sci-Hi-C libraries were generated from five human cell lines (Table 1), which are available via the 4D Nucleome data portal (http://4dnucleome.org) [16]. Each sci-Hi-C library consists of single cells from one cell type or a mixture of cells from two different cell types, as explained in Ramani *et al*.

**Table 1:** Summary statistics of all nine sci-Hi-C libraries.

Library name	Reads		# of cells	Contacts				
	Total	Uniquely mapped		Total	Cis (%)	Trans (%)	Median	Mean
**H1Esc.R1**	170,540,464	17,968,958	1,863	6,460,047	76.84	23.16	2,210	3,468
**H1Esc.R2**	120,575,805	21,552,922	4,199	20,068,026	76.55	23.45	2,887	4,779
**H1Esc-HFF.R1**	142,008,139	26,180,611	1,931	19,411,330	82.97	17.03	4102	10,052
**H1Esc-HFF.R2**	168,117,805	22,894,572	1,583	15,726,319	82.28	17.72	4,476	8,262
**HFF-GM12878.R1**	298,427,055	32,821,460	2,312	19,102,636	79.91	20.09	4,949	8,262
**HFF-GM12878.R2**	206,197,128	27,325,538	2,910	24,532,338	80.36	19.64	5,192	8,430
**GM12878-IMR90.R1**	119,664,770	4,640,327	590	4,422,045	82.06	17.94	6,408	7,495
**IMR90-HAP1.R1**	31,741,750	8,604,353	2,002	8,348,732	91.55	8.45	3,185	4,170
**IMR90-HAP1.R2**	191,450,674	21,990,859	1,998	21,769,917	91.73	8.27	7,864	10,896

The total number of reads indicate total number of sequenced reads, whereas uniquely mapped reads indicate deduplicated uniquely mapped reads (MAPQ >= 30) that are associated with their barcodes and before filtering out low quality cells. The number of cells and contact counts are computed after processing the libraries as described in Ramani *et al*. The median and mean contact counts are reported here per cell.

#### Cell culture

Human embryonic stem cells (hESC) H1, HFF-hTERT clone #6 (HFFc6), GM12878, IMR90 and HAP1 cells were obtained through the 4D Nucleome Consortium and cultured following the respective protocols standardized by the 4D Nucleome Consortium (4DN SOPs, https://www.4dnucleome.org/cell-lines.html).

#### Sci-Hi-C library construction

Sci-Hi-C libraries were generated as previously described in Ramani *et al*. [3] with some modifications. Briefly, cells grown at the exponential phase were fixed by incubating with formaldehyde at room temperature (25°C) for 10 min. The final concentration of formaldehyde was 2% for HFFc6, GM12878, IMR90 and HAP1 cells, and 3.5% for H1ESCs. Crosslinking was quenched by incubating with 0.125 M glycine on ice for 15 min. Crosslinked cells were then permeabilized with their nuclei intact and subjected to DpnII digestion to fragment the chromatin. Nuclei were then distributed to 96 wells, wherein the first-round barcodes were introduced through ligation of barcoded biotinylated double-stranded bridge adaptors. Intact nuclei were then pooled and subjected to proximity ligation, followed by dilution and redistribution to a second 96-well plate (no more than 25 nuclei per well). Crosslinking was reversed by incubating the nuclei in 2x NEBuffer #2 at 65°C overnight, followed by digestion with restriction enzymes Alu I and Hae III at 37°C overnight. After dA-tailing, second round barcodes were introduced through ligation of barcoded Y-adaptors. A total of 0.8 volume of Ampure beads were then added to each well, and all reaction samples in the 96-well plate were once again pooled and purified, and biotinylated junctions were then purified with Dynabeads M-280 streptavidin beads. Illumina sequencing libraries were amplified by PCR (13–15 cycles were used for each 96-well sample) and purified by two rounds of 0.8 volume Ampure beads. Libraries were sequenced by 2 x 250 bp paired-end run on a HiSeq-2500.

#### Data processing

The sci-Hi-C libraries were processed according to the protocol described in Ramani *et al*. [3]. In summary, the outer and inner sci-Hi-C barcodes from the demultiplexed Illumina reads were identified and assigned by comparing against the known 96 barcode sequences with a Levenshtein distance cutoff of 2, discarding read pairs that did not have matching outer or inner sci-Hi-C barcodes. The processed read pairs were mapped to the human genome assembly hg19 using Bowtie2 2.2.3 [17] with default settings. The aligned reads were filtered with MAPQ ≥ 30, and the closest DpnII site to each read was determined with bedtools [18] and HiC-Pro [19]. The filtered reads were then deduplicated and deconvolved into cellular indices. As in previous work, cells with fewer than 1000 unique reads, cis/trans-chromosomal contact ratio lower than 1, or uniquely mapped read percentage lower than 95% were filtered out. After this mapping step, the data covers 19,388 cells with a median coverage of 4019 sci-Hi-C contacts per cell. The sci-Hi-C contacts for each cell were aggregated into a square contact map using bins of 500 kb. The sci-Hi-C contacts for each cell were binned into a matrix representation using bins of 100kb, 250kb, 500 kb, and 1Mb.

For the single-cell Hi-C and snHi-C datasets generated by Nagano *et al*. and Flyamer *et al*., respectively, we used mapped and processed Hi-C interactions as provided by the authors.

#### Downsampling of single cell Hi-C data

To control the effects of variation in the number of single cell Hi-C interactions per cell, we performed downsampling to bring the number of Hi-C interactions per cell to same or similar levels. Downsampling involves a simple sampling without replacement strategy where we select *N* Hi-C interactions from a set of total *M* Hi-C interactions for cell *K*, where *N < M*. The resulting subset of downsampled interactions are binned into contact matrices as described in the previous section.

### ScHiCluster

ScHiCluster was installed from GitHub (https://github.com/zhoujt1994/scHiCluster) and applied to our datasets binned at 500kb resolution with default settings. To filter out low coverage cells as Zhou *et al*. recommended in their publication, we discarded cells with non-diagonal intra-chromosomal contact count less than 5000 contacts per cell and intra-chromosomal non-diagonal contact count less than *x*_*i*_, which is the length of chromosome *i* in Mb.

### Topic modeling

Latent Dirichlet allocation is used to decompose the cell-locus pair (LP) matrix into cell-topic and topic-LP distributions, representing cells as a mixture of topics that contain specific sets of locus pairs. In short, LDA assumes that a “document” (i.e., data corresponding to a single cell) is generated by the following steps: (1) choosing a topic mixture for the document according to a Dirichlet distribution over some specified number of topics, and (2) generating words in the document by iteratively sampling a topic from the topic mixture and selecting a word from the topic’s multinomial distribution [20]. With this generative model, the LDA identifies a set of topics that have most likely generated a given collection of documents.

To apply topic modeling to sci-Hi-C data, we used a customized version of the cisTopic package (v 0.3.0) in R [15]. As in their cisTopic model, we treated cells as documents, but in our case the role of words is played by genomic locus pairs (LPs), rather than genes. The cell-LP matrix was built from the binned single cell contact matrix files, in which the contact matrices were converted to vectors, concatenated, and binarized.

We used the collapsed Gibbs sampling approach to run LDA in cisTopic, in which the topic assignment of each locus pair in each cell is drawn from a conditional multinomial distribution [20]. Specifically, we used the ‘runCGSModels’ function with default settings. For our analysis, we considered intra-chromosomal locus pairs that are within 10 Mb of one another. With sci-Hi-C data binned at 500 kb resolution and only considering the contacts in autosomal chromosomes (chr 1–22), this range includes 111,340 locus pairs. In our data, these locus pairs receive 83.8% of the observed intra-chromosomal contacts.

#### Model selection

We ran LDA on our datasets with different numbers of topics (5-135). We considered two metrics to determine the optimal range of topic numbers: the topics that maximized the log-likelihood value of the model and the average Jensen-Shannon divergence of normalized topic contribution values of LPs between all pairs of topics [21, 22]. For our dataset, we concluded that the optimal number of topics was in the range 30-90. We assessed the clustering of the cell types in embedding of the topic-cell matrix and selected the number of topics that produced the highest average Silhouette coefficient (30).

### Dimensionality reduction

For baseline method comparison, the cell-locus pair matrix was reduced to 50 dimensions by performing PCA [7] and then was further reduced to two dimensions with UMAP [9] after removing the first principal component.

For embedding the topic modeling and scHiCluster results, UMAP was applied directly to the cell-topic matrix (for topic modeling) and directly to the PCA-reduced imputed single cell contact matrices as described by Zhou *et al*., after removing the first principal component to remove the coverage effect (for scHiCluster).

### Silhouette analysis

Silhouette analysis measures how close a single cell is to all other single cells of the same cell type versus the closest cell from another cell type [23]. To evaluate the clustering performance of topic modeling, we computed an average silhouette coefficient for each cell type from the UMAP embedding of the cell-topic matrix using the cell type labels as cluster assignments. The silhouette coefficient for a cell *i* is defined as

$$\begin{array}{c} \text{Silhouette}\;\text{coefficient}\left(i\right)=\frac{p(i)-q(i)}{\text{max}(p(i),q(i))} \end{array}$$

where *p*(*i*) is the minimum average inter-cluster distance between cell *i* and all the other cells with different cell type labels, and *q*(*i*) is the average intra-cluster distance between cell *i* and all the other cells with the same cell type labels. The silhouette coefficient ranges from −1 to 1, in which a value close to 1 indicates that the cells of the same cell type are well clustered.

### Topic specificity

#### Topic to cell type assignment

The two-sample Wilcoxon test determines whether two empirical distribution functions significantly differ from one another [24]. We conducted one-sided, two-sample Wilcoxon tests on the distributions of normalized topic contributions from two different cell types to determine cell type specificity of discovered topics. To normalize the topic contributions, the topic assignments in each cell were divided by the total topic assignments for each topic. The normalized topic contributions were then grouped by cell types to produce a distribution. Since there are a total of five cell types in our datasets, we conducted four separate pairwise Wilcoxon tests for each cell type pair for each topic, to ensure that the topic is specific to only one cell type. For example, to test whether topic 1 is specific to GM12878, we performed the one-sided Wilcoxon test on the topic 1 probability distributions of H1Esc and GM12878, HFF and GM12878, IMR90 and GM12878, and HAP1 and GM12878. A topic was assigned to a cell type if the Benjamini-Hochberg (BH) adjusted p-values for all four tests were below the significance level of 0.01. The cell type-specific topics were further filtered out if the ratio of average normalized topic contribution value for the assigned cell type and the second highest average normalized topic contribution value for the other cell type was higher than 1.5. This filter was used to increase the specificity of topic assignment to cell types.

#### Locus pairs to topic assignment

To assign locus pairs to each topic, we follow the approach described in González-Blas *et al*. First, locus pair topic contribution values were normalized by dividing by the total locus pair contributions for each topic. The normalized topic-LP matrix was then converted to probabilities of observing a locus pair in a topic, with the formula:

$$\begin{array}{c} P_{l,t}=\beta_{l,t}(\text{log}\;\beta_{l,t}-\sum_{t=1}^{T}\frac{log\beta_{l,t}}{T}) \end{array}$$

where *P*_*i*,*t*_ is the probability, *β*_*l*,*t*_ is the normalized contribution value for locus pair *l* and topic *t*, and *T* is the total number of topics in the model [15]. The LP topic probabilities were then min-max normalized.

We then fitted a gamma distribution to the normalized locus pair topic contribution values. Using a cutoff threshold value that reflects the 99.75th percentile of the fitted distribution, we assigned locus pairs that had normalized contributions values higher than this threshold.

### Compartment analysis

#### Compartment calling

Bulk Hi-C data for H1Esc [16], HAP1 [25], HFF [16], and IMR90 [26] cells and aggregated sci-Hi-C data for GM12878 cells were used to assign A/B compartments. Each intra-chromosomal matrix was binned at 500kb resolution and was normalized using Iterative Correction and Eigenvector decomposition (ICE) [27] followed by removal of the genomic distance effect. The spearman correlation matrices were computed from the ICE normalized intra-chromosomal matrices and decomposed into eigenvectors. The first and second eigenvectors, along with gene density, were used to assign compartments to each genomic bin, similar to what was done by Lieberman-Aiden 2009.

#### Locus pair compartment structure analysis

Using the A/B compartment calls obtained from bulk Hi-C and aggregated sci-Hi-C datasets, each locus pair was assigned as AA, AB, or BB, based on the cell type-specific compartment assignments at the two ends of the locus pair. We then computed the observed proportion of each compartment label among LPs associated with each cell type-specific topic. To ascertain which particular compartments are enriched in each topic, we estimated the expected proportion of each compartment using the compartment labels of all topic-associated LPs and compared the observed proportions with the expected to measure enrichment of LP compartment structures (S14A and S14B Fig). We assessed the significance of differences in the expected and observed proportions using a chi-square test for independence at the significance level of 0.01 with BH FDR correction. In our analysis, every topic exhibited significantly different locus pair compartment composition from the expected, demonstrating an enrichment of either AA, AB or BB compartment assignment.

#### Compartment switch analysis

To obtain compartment switching regions, we compared compartment assignments across cell types and filtered for regions with a different compartment assignment in one cell type versus the compartment assignments for other four cell types. We refer to these regions as compartment switching regions.

Using these regions, we computed the proportion of locus pairs with the switching regions in the cell type-specific topics (observed) and compared with the proportion observed in all topics (expected). Chi-square tests were performed between the expected and observed proportion of locus pairs with compartment switching regions. Similarly, we computed the enrichment of A-to-B and B-to-A switching regions in the cell type-specific topics. The observed and expected proportions were divided and transformed to log2 scale.

### Processing and analysis of RNA-seq data

Bulk RNA-seq data for GM12878, H1Esc, and IMR90 cells (two biological replicates each) generated by the Gingeras lab at CSHL were downloaded from ENCODE [28] (https://www.encodeproject.org). Two technical replicates of RNA-seq data for HFFc6 cells were obtained from the 4DN Data Portal (https://data.4dnucleome.org, Yue lab) and one biological replicate of RNA-seq data for HAP1 cells from the publication by Gowen *et al*. [29].

The RNA-seq datasets were aligned to the human genome hg19 using STAR (v2.7.3a) [30] with settings standardized by ENCODE. featureCounts (v2.0.1) [31] software was then used to count the number of fragments that are associated with exons in each aligned data. Differential gene expression tests were performed with DESeq2 (v1.24.0) [32] software, by fitting each gene with a general linear model with the cell type identity as independent variables.

Each set of upregulated cell type-specific genes (Wald test, q-value < 0.01 and log2 fold ratio > 2) was mapped to the 500kb genomic bin they fall into. Compartment switching regions that encompass the differentially upregulated genes were then selected and used to compute the enrichment of B-to-A transitions against the frequency of B-to-A transitions in cell type-specific compartment switching regions that occur genome-wide.

## Results

### Applying topic modeling to sci-Hi-C datasets

**Fig 1:**
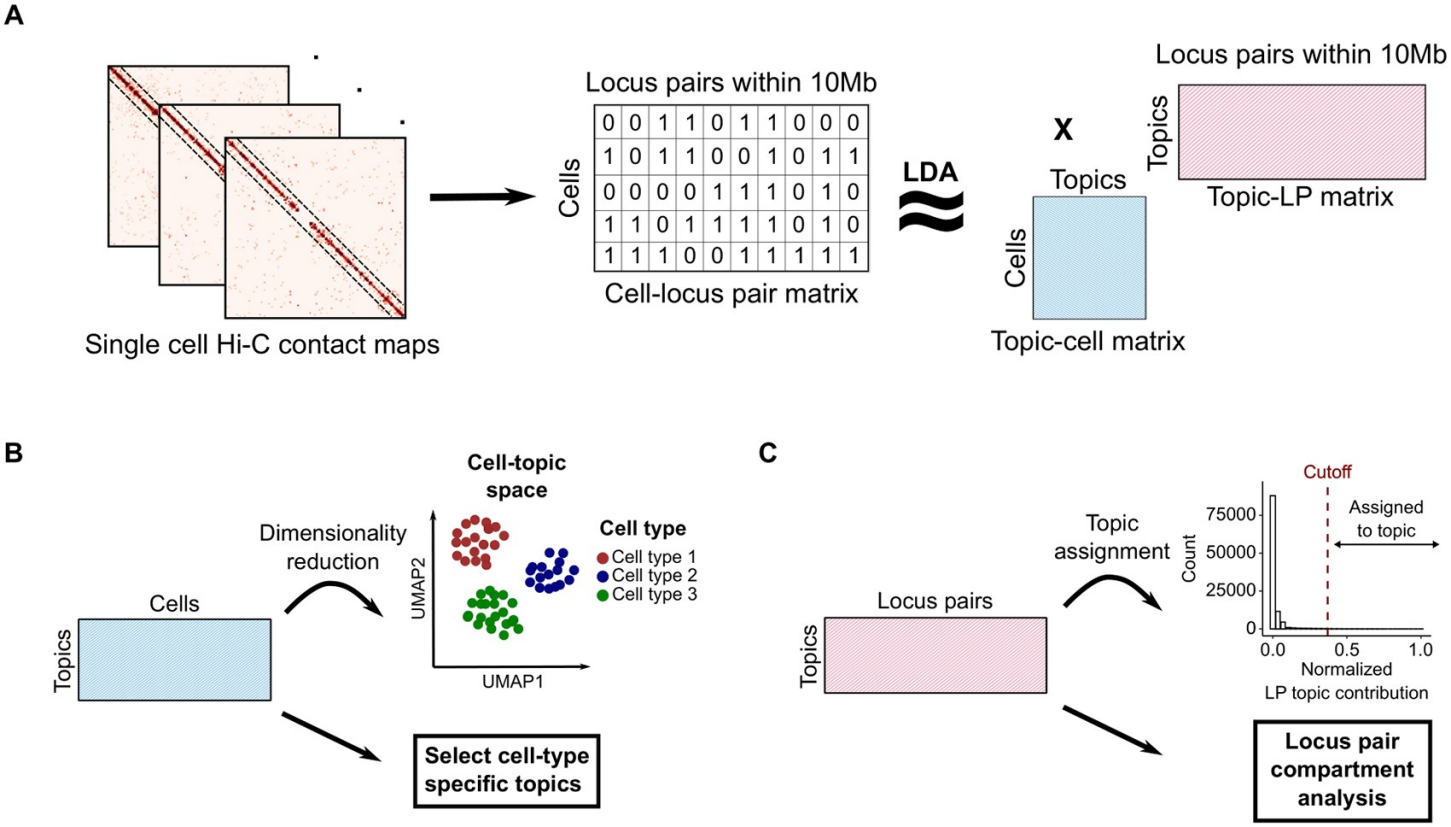
Topic modeling of sci-Hi-C data. (A) Sci-Hi-C data was binned at 500kb resolution and processed into a cell-by-locus pair (LP) matrix. The resulting cell-LP matrix was provided as input to LDA to produce cell-topic and LP-topic matrices. Only intra-chromosomal contacts spanning distances <10 Mb were considered for analysis. (B) The output cell-topic matrix is projected into 2D using UMAP and can also be used to select cell type-specific topics. (C) The output LP-topic matrix can be used for selecting a subset of locus pairs that are most representative of each topic and for performing locus pair compartment comparison.

We used Latent Dirichlet allocation (LDA) based topic modeling to decompose our sci-Hi-C data into a collection of topics (Fig 1). First, we binned the intra-chromosomal sci-Hi-C matrices at 500kb resolution and included locus pairs (LPs) that are made within 10Mb, representing the data in a matrix with 19,388 row (cells) and 111,340 columns (locus pairs). Because a majority of the *cis*-contacts are concentrated at 10Mb range (S1 Fig), we reasoned that only considering the locus pairs within this range strikes a balance between data sparsity and model complexity. Including LPs separated by longer distances marginally increases the amount of information being used, while increasing the number of model parameters dramatically. We further investigate the effects of resolution and distance range on model training and performance in following sections.

Using the binarized cell-LP matrix as an input, we trained our LDA model over a range of topic numbers. We narrowed down to find the range of optimal topic numbers that included topics that maximized the log-likelihood value of the model and the average pairwise Jensen-Shannon divergence of locus pair (LP) contribution values between each topic [21, 22] (S2 Fig). Based on the models’ performances of clustering of cell types, we selected the value 30 to be the optimal topic number, yielding a 19, 388 × 30 cell-topic matrix and a 111, 340 × 30 LP-topic matrix.

**Fig 2:**
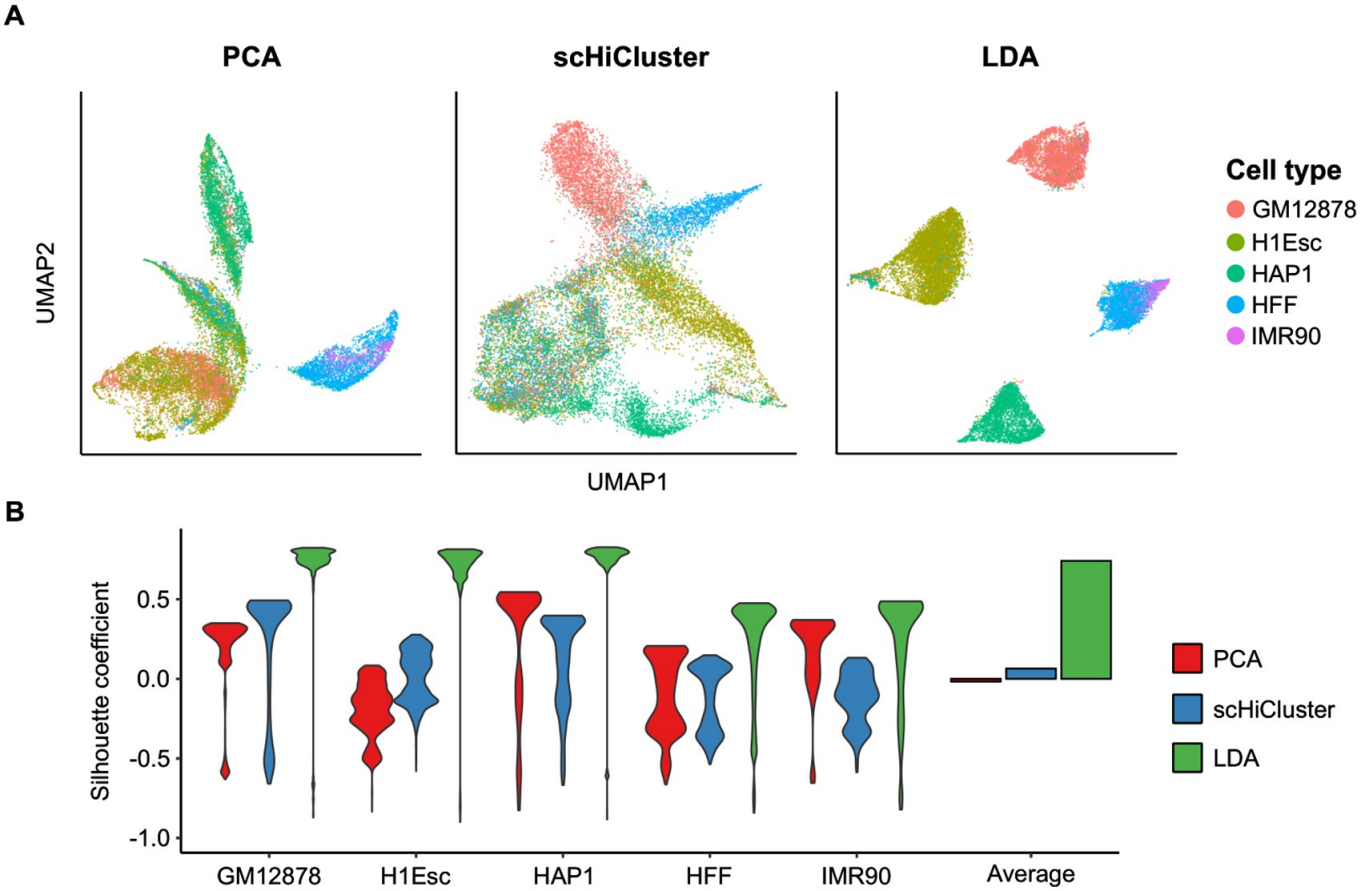
Comparison clustering performance of topic modeling and other methods on our sci-Hi-C data. (A) PCA, scHiCluster, and LDA were performed on the sci-Hi-C contact matrices and the dimensionality reduced data was embedded using UMAP. (B) Silhouette coefficients computed from the UMAP embeddings for each cell type.

To evaluate whether topic modeling successfully extracted biological information from the sparse sci-Hi-C data, we applied UMAP [9] to the resulting cell-topic matrix (Fig 2A). Most notably, the data exhibits strong clustering of cells by their cell types, suggesting that cells of the same cell type have similar topic contribution profiles. An exception is HFF and IMR90 cells, which are embedded close together, likely reflecting the fact that they are both fibroblasts (foreskin and fetal lung, respectively).

We also investigated the effect of potential confounders on the learned embedding. Visualization of the data with color according to their library ID shows little evidence of batch effects (S3 Fig). In addition, training the LDA model on data that was downsampled to the same coverage levels led to a similar separation of cell types in the UMAP space with similar clustering performance (S4 Fig). Specifically, downsampling was done by computing the median contact count (2751 contacts) across the 111,340 proximal locus pairs, discarding cells with fewer than the median number of contacts, and randomly downsampling the remaining 9697 cells to median number of contacts. Within each cell type, we observe some clustering by coverage (S5 Fig), suggesting that the effect of coverage on the learned model is small relative to the differences between cell types.

### Evaluating the performance of topic modeling on cell type clustering

We compared the performance of topic modeling against two methods: PCA as a baseline and the scHiCluster method [12] (Fig 2A). Applying topic modeling to our sci-Hi-C datasets significantly improved the clustering of cells by their cell types compared to the other methods, with an average silhouette coefficient of 0.741 (Fig 2B). The average cell type-specific silhouette coefficients computed from the cell-topic matrix were higher in all cases, suggesting that topics contain cell type-specific information. Due to the imputation step requiring a high number of contacts in each intra-chromosomal matrix, scHiCluster failed to properly cluster cell types without filtering out relatively low coverage cells (< 5000 non-diagonal contacts per cell) in the datasets. After filtering out low coverage cells following the criteria described by Zhou *et al*., scHiCluster performed as well as LDA (S6 Fig). However, the filtering process removed 88.4% of the cells in the dataset, which renders a large number of cells unusable for clustering and other subsequent analyses when dealing with sparse sci-Hi-C datasets. Topic modeling had superior performance independent of the filtering process, which highlights the robustness of topic modeling on sparse single-cell Hi-C datasets.

### Identifying and characterizing cell type-specific topics

**Fig 3:**
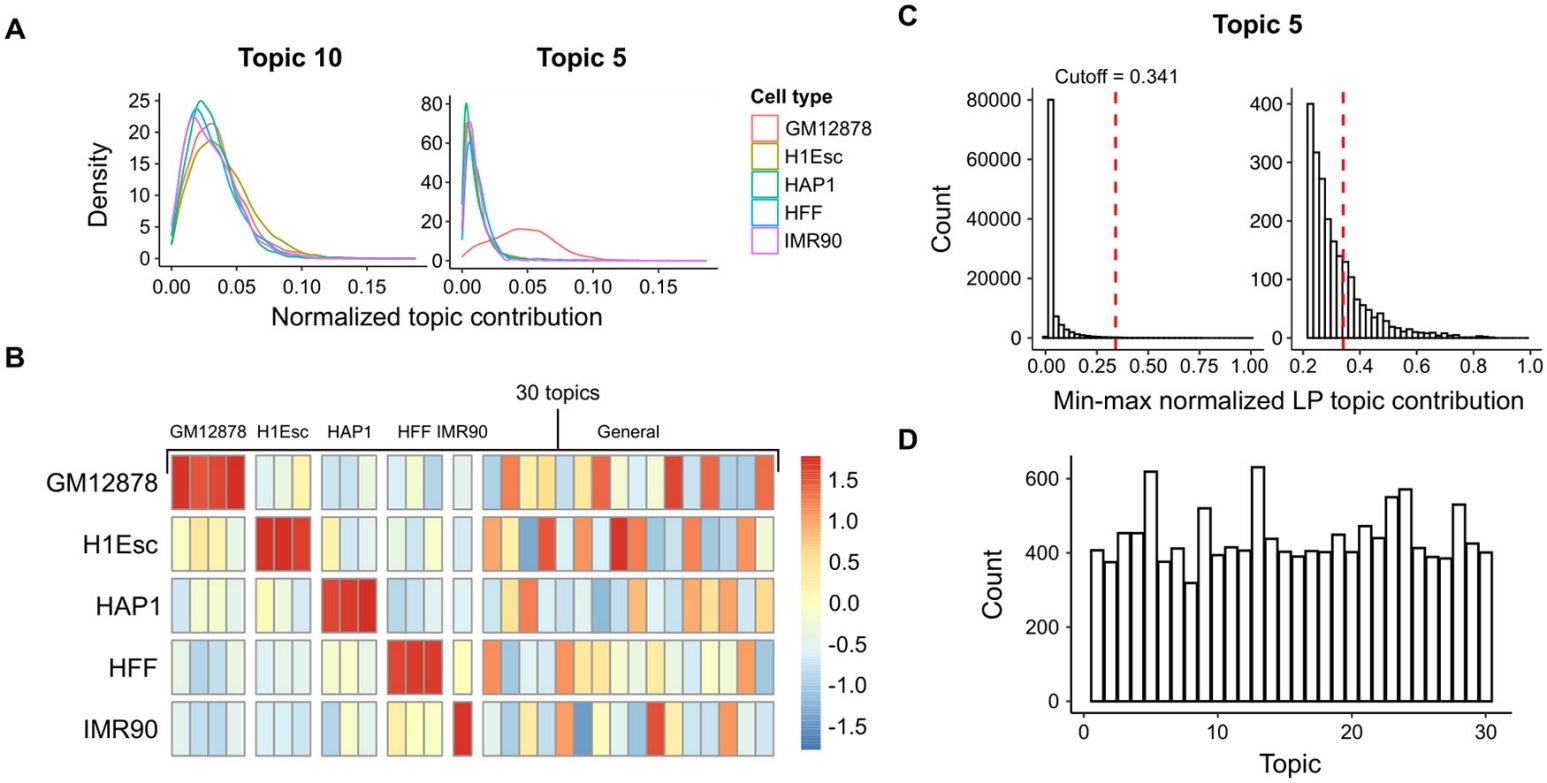
Assignment of topics to cell types and locus pairs. (A) Distributions of normalized topic contribution values for general (left) and GM12878-specific (right) topics, colored by cell type. (B) Heatmap of Z-scaled average normalized topic assignment values for each cell type. Columns are annotated by the specificity of the topics. (C) Histogram of min-max normalized LP topic contribution values for GM12878-specific topic 5. The cutoff threshold value was determined by fitting gamma distribution to the values and taking the 99.75th percentile. (D) Barplot showing the number of locus pairs assigned to each topic.

Next, we sought to better understand the properties of the learned model. Visualization of the cell-topic matrix (S7 Fig) and the heterogeneity in topic contributions across cell types (Fig 3A) suggested that many of the topics are highly associated with particular subpopulations of cells. We performed pairwise two-sample one-sided Wilcoxon tests on the distributions of normalized topic contribution values to identify topics that are specifically enriched for each of the five cell types. To further increase the specificity of the topic to cell type assignment, we only considered topics with average normalized topic assignment value for the assigned cell type to be higher than 150% of the average normalized topic assignment value in other cell types. With this approach, we assigned a total of 4, 3, 3, 3, and 1 topics specific to GM12878, H1Esc, HAP1, HFF, and IMR90, respectively.

We then identified locus pairs that are strongly associated with each topic. Similar to the normalizing approach described in González-Blas *et al*., locus pair topic contributions were converted to probabilities then min-max normalized. The normalized topic-LP probabilities were fitted to a gamma distribution and locus pairs with normalized probabilities in the top 99.75% percentile of the fitted distribution were assigned to each topic (Fig 3C). With this approach, we associated a median number of 412 locus pairs per topic (Fig 3D).

### Applying topic modeling to datasets binned at different resolutions and with inter locus pair distance

We explored how the resolution of the input contact matrices and the inter locus pair distance affect the performance of topic modeling. First, we applied topic modeling to sci-Hi-C matrices binned at 100kb, 250kb, 500kb, and 1Mb resolution. At 100kb resolution, accurate cell type clustering was achieved only when the model was trained with a small number of topics (5), most likely due to a significant increase in sparsity (S8A Fig). At higher topic numbers, the model produced severe artifacts, which can be seen in the embeddings of the topic-cell matrices (S8A Fig). These artifacts were present even though the model was trained with a relatively small number of topics (10), suggesting that 100kb is not an appropriate resolution for our datasets. The performance of LDA at 250kb resolution was similar to that of 500kb resolution in that the model was able to detect cell type-specific topics and cluster cells by cell types in the UMAP reduced space (S8B and S9 Figs). At a more coarse 1Mb resolution, the clustering of cells was slightly worse than that of 250 and 500kb resolution.

In addition, we varied the inter locus pair distance and number of locus pairs to include in the topic model, while holding the resolution of the matrices constant at 500kb. Even with a topic-cell matrix that only includes locus pairs within 3Mb of one another, the model was sensitive enough to separate cells by their cell types (S10 Fig). Including locus pairs spanning 15 and 20 Mb, respectively, did not markedly improve the clustering of the cell types in the topic reduced space, suggesting that going beyond the 10Mb range does not add more information to help distinguish the cell types (S11 Fig).

Collapsed Gibbs sampling based LDA method is easily scalable, allowing the method to be easily applied to large single cell Hi-C datasets over a range of topics. Even though the computational time and resources required to train the topic model with 50 topics exponentially increases at finer resolution (S12 Fig), training the model with the datasets binned at 100kb resolution only took approximately 28 hours and 11Gb of memory.

### Applying topic modeling to published single cell Hi-C datasets

#### Capturing cell cycle effects with topic modeling

We asked whether topic modeling is able to capture the changes in the three dimensional organization of the genome during cell cycle stages. We observed a group of cells in the UMAP projection of sci-Hi-C data, that was composed of cells from all five cell types (S13A Fig). The cells in this cluster had higher percentages of long-range mitotic contacts (2–12Mb) and lower percentages of short-range contacts (< 2Mb) (S13B–S13D Fig), suggesting that topic modeling learns cell cycle effect related topics. However, a large majority of cells in the sci-Hi-C datasets were collected during interphase, thus we did not find this dataset suitable for studying the cell cycle effects on the 3D organization of chromatin.

**Fig 4:**
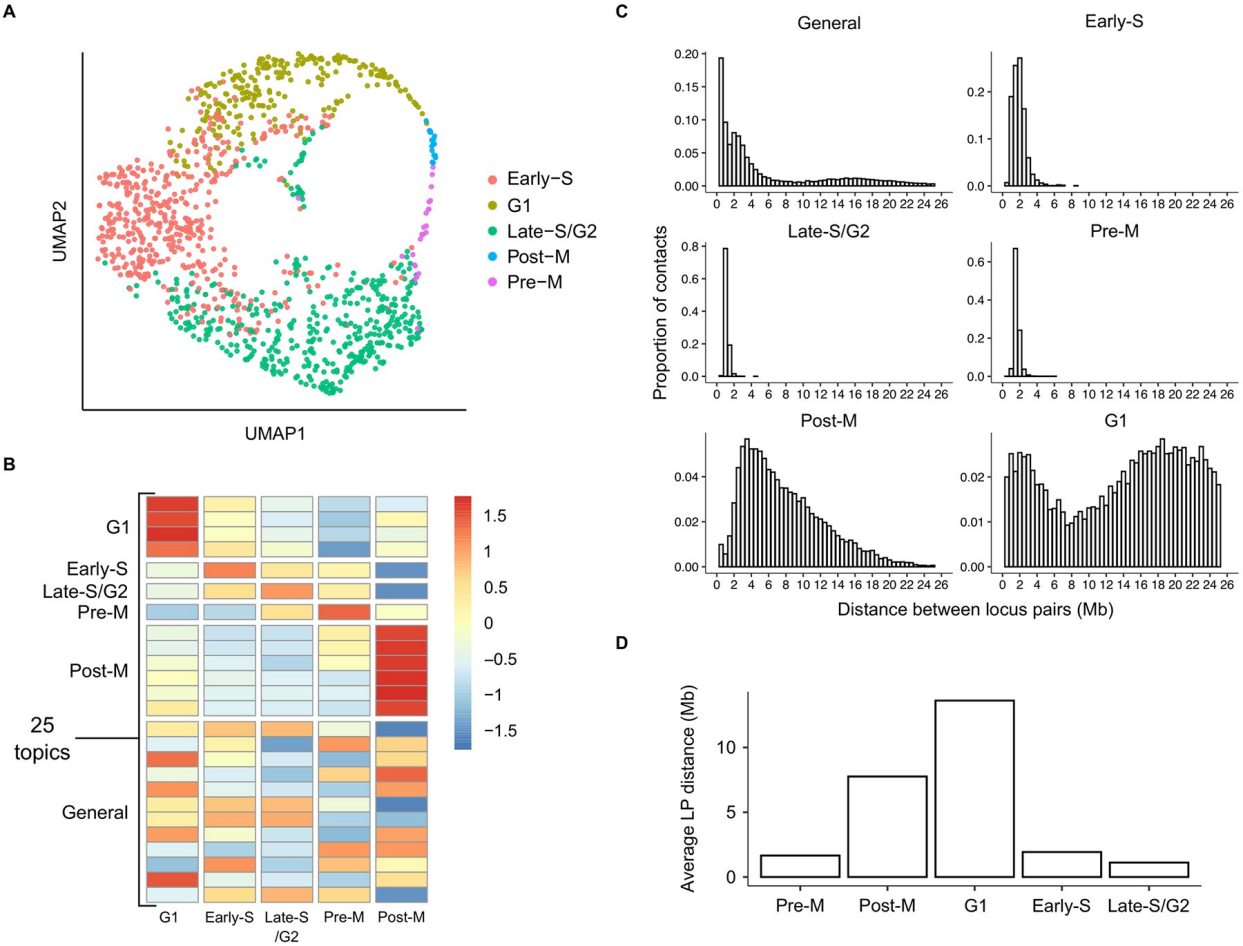
Cell cycle effect is captured by topic modeling. (A) Two-dimensional UMAP projection of topic-cell matrix obtained by applying LDA to Nagano *et al*. data. (B) Heatmap of Z-scaled average normalized topic assignment values. Rows are annotated by the specificity of the topics. (C) Barplot showing the distribution of inter locus pair distances in the cell cycle stage-specific and general topics. (D) Average LP distances in topics specific to each cell cycle stage group.

To further explore this hypothesis, we applied topic modeling to the Nagano *et al*. dataset, which consists of single cell Hi-C data collected from mouse embryonic stem cells at different cell cycle stages [2]. Unlike the sci-Hi-C dataset, we considered locus pairs within 25Mb of another, because we wanted to include long-range contacts that are prominent in cells in mitotic stages [2, 33]. The embedding of the learned topic-cell matrix showed ordering of cells by cell cycle phases (Fig 4A), with at least one or more topics specific to each cell cycle stage (Fig 4B). We then investigated whether the distributions of inter locus pair distances in the cell cycle-specific topics correlate with contact decay profile changes during the cell cycle [2]. Similar to the results from Nagano *et al*., topics specific to early and late S phases were enriched for LPs separated by shorter distances, with an increase in long range interactions during mitosis (Fig 4C). The locus pair distances observed in G1 phase-specific topics were the longest (Fig 4D), indicating that a higher number of long-range contacts are made in cells at the G1 stage.

#### Applying topic modeling to Flyamer *et al*. data

**Fig 5:**
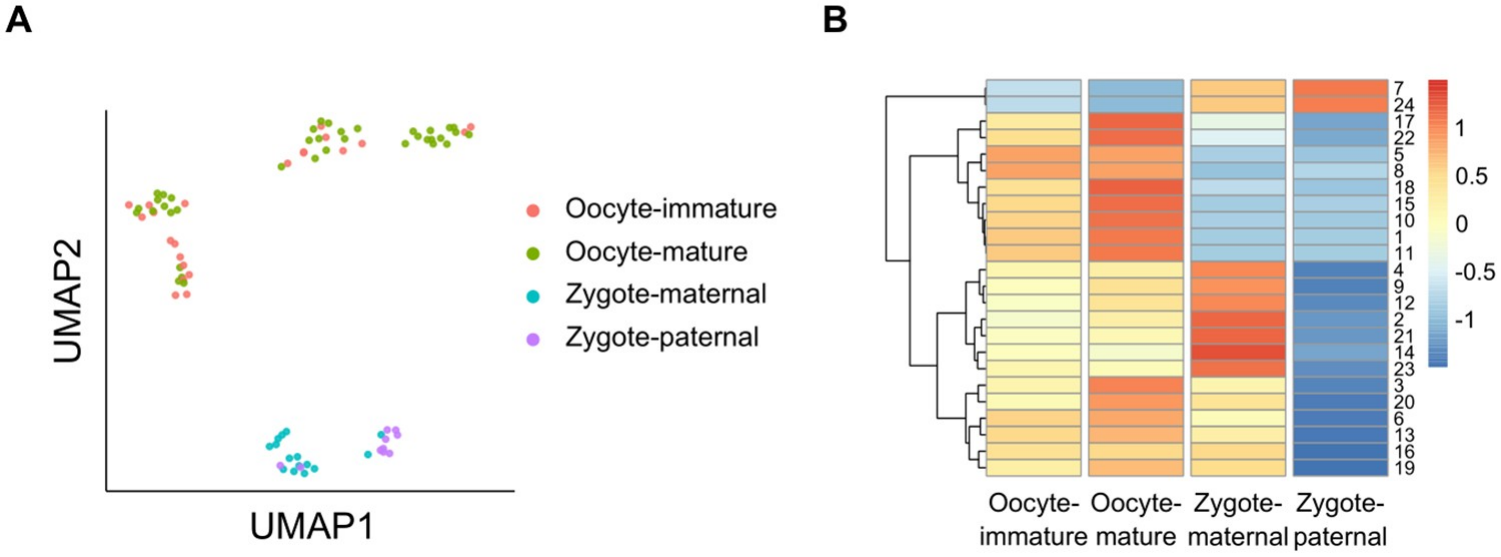
Topic modeling discovers topics specific to oocytes and zygotes. (A) Two-dimensional UMAP projection of topic-cell matrix obtained by applying LDA to the randomly downsampled Flyamer *et al*. data (n = 85 cells). The bottom 50% of the cells were discarded to mitigate the coverage effects. (B) Heatmap of Z-scaled average normalized topic assignment values from the downsampled data. Rows are annotated by the specificity of the topics.

We applied LDA to the Flyamer *et al*. single nucleus Hi-C data derived from mouse oocytes and zygotes [6]. Due to skewness in coverage that ranges from 1990 to 2.1 million contact counts per cell, in addition to the low number of cells in the data (∼170 cells), the coverage of each cell had a strong effect on the training of the topic model (S14 Fig). After downsampling the contact counts to the median and discarding the bottom 50% of the cells, we were able to train the model to discover topics that were specific to oocytes and zygotes. The method also successfully segregated oocytes, maternal zygotes, and paternal zygotes (Fig 5A). Although the immature and mature oocytes were mixed together in the UMAP embedding and shared similar topic structure, the topic contributions of the shared topics were generally stronger in mature oocytes (Fig 5B).

### Examining the compartment structure of cell type-specific topics

To understand what each cell type-specific topic captures in the sci-Hi-C data, we examined the Hi-C compartment structures of the locus pairs that characterize each topic. Previous analysis of bulk Hi-C data suggests that chromatin can be usefully segregated into two compartments, A and B, where the A compartment is associated with euchromatin and the B compartment with heterochromatin [34]. Because these compartment structures vary by cell type [34, 35], we hypothesized that cell type-specific topics might be enriched for locus pairs that exhibit cell type-specific compartment structures.

#### Evaluating topic-associated locus pair compartment structure within cell type

First, we assessed whether certain locus pair compartment structures are overrepresented in the cell type-specific topics. Using the A/B compartment calls for each cell type, each locus pair was assigned one of three labels: (1) both loci are in the active, more euchromatic compartment state (AA), (2) both loci are in the inactive and more repressive compartment state (BB), or (3) one locus is active and the other inactive compartment state (AB). The frequency of each locus pair compartment label in each cell type-specific topic was compared against that of locus pairs in all topics to assess the enrichment of any compartment labels (S15A Fig).

This analysis showed that each cell type-specific topic contains LPs that are exclusively enriched for AA and BB but depleted in AB compartments (S15B Fig). Interestingly, even though the proportion of locus pairs in AA and BB compartment configurations is approximately equal across all topics, most of the cell type-specific topics were enriched for one of these compartments (S15C Fig). As expected, the number of topics enriched for AA and BB compartments varied from cell type to cell type, with IMR90 specific topics highly enriched for AA compartments. Note that IMR90 cells in our datasets were the least abundant and had the lowest coverage (S16 Fig); therefore, only a small number of topics and locus pairs were assigned to IMR90. This may explain why we only have IMR90-specific topics that are enriched for the AA compartment.

#### Investigating compartment switching regions in topic-associated locus pairs

**Fig 6:**
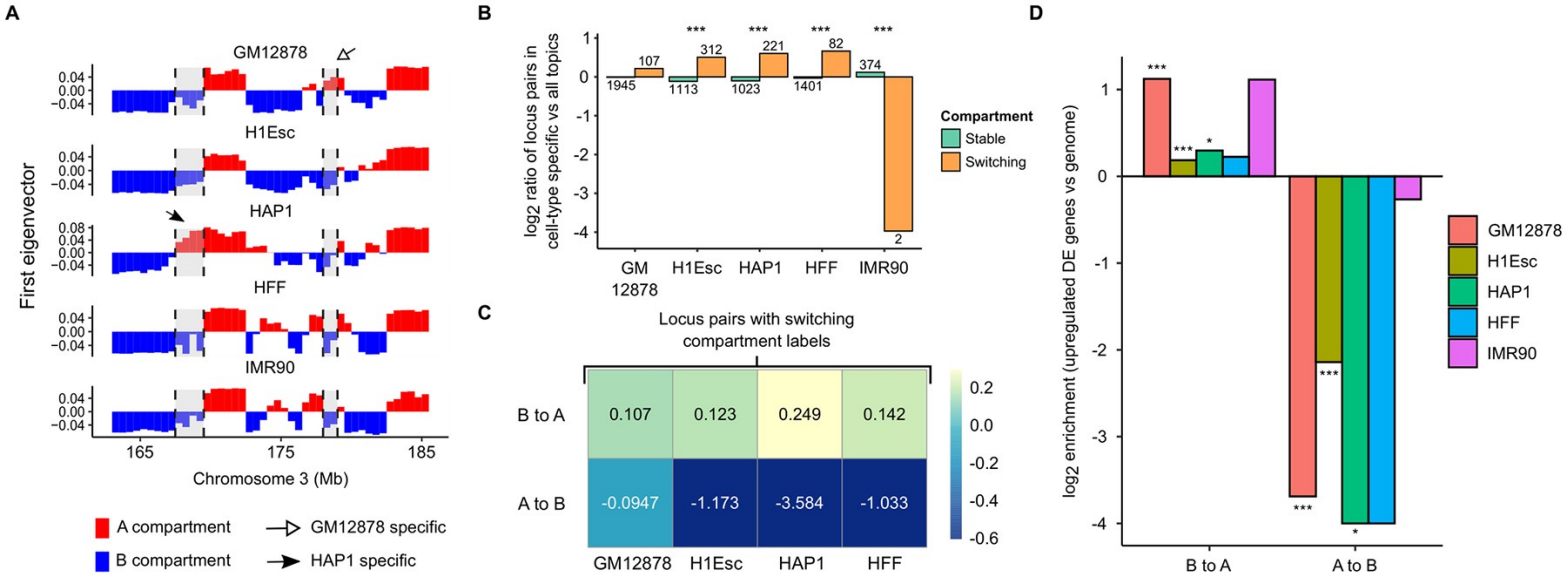
Locus pairs in cell type-specific topics are enriched in compartment switching regions. (A) Compartment calls for each cell type were compared to obtain compartment switching regions. Compartment switching regions (B-to-A) in chromosome 3 of HAP1 (left) and GM12878 (right) are highlighted. (B) Barplot showing log2 enrichment of locus pairs with compartment switching and stable regions in cell type-specific topics vs all topics. The numbers below and above the bars represent the number of compartment switching regions. P-values by chi-square test: *** < 0.001. (C) Heatmap of log2 enrichment of compartment transitions of regions that are in cell type-specific vs. all topics. (D) Barplot of log2 enrichment of A/B compartment dynamics in regions that contain upregulated cell type-specific differentially expressed (DE) genes vs. the genome. P-values by Fisher’s exact test: * < 0.05, ** < 0.01, *** < 0.001.

Furthermore, we investigated whether the cell type-specific topics are enriched in cell type-specific compartmental organization. In our analysis, we defined a compartment switching region as a region at which the compartment label in one cell type is different from other cell types (Fig 6A). Since these compartment switching regions are unique to each cell type, they suggest that topic modeling is discovering topics related to Hi-C contacts resulting from cell type-specific compartmental organization.

We compared the observed proportion of locus pairs with one or more compartment switching regions in cell type-specific topics and the expected proportion of locus pairs in all topics. Strikingly, we observed an enrichment of contacts made with at least one compartment switching region in cell type-specific topics across all cell types except IMR90, supporting our hypothesis that topic modeling identifies cell type-specific compartment structures (Fig 6B). Again, the small number of contacts with compartment switching regions in IMR90-specific topics is likely due to the dearth of IMR90 data. Conversely, there was a depletion of locus pairs in topics that were not specific to the cell types (S17A Fig).

Next, we examined which compartment transitions were mostly occurring in these switching regions. With the exception of IMR90-specific topics, which only consisted of two locus pairs with A-to-B compartment transitions, there was an enrichment of B-to-A transitions in other cell type-specific topics (Fig 6C). In contrast, the proportion of compartment transitions identified in non cell type-specific topics did not deviate much from the expected proportion, with a slight enrichment of A-to-B (S17B Fig). Altogether, these results suggest that the learning of topics important in distinguishing cell types in topic modeling is likely driven by the intra-chromosomal contacts made with repressive to active compartment transitioning regions.

#### Associating cell type-specific B-to-A compartment transition to marker gene expression

Previous studies [36, 37] have shown an enrichment of B-to-A compartment reorganization from one cell type to another in regions with upregulated genes. Therefore, we asked if the B-to-A transitions were enriched in regions containing upregulated marker genes associated with the cell types in our datasets. Using the bulk RNA-seq data available from multiple sources [16, 28, 29], we obtained a set of upregulated cell type-specific genes for each cell type. Similar to the previous findings, the cell type-specific genes were overrepresented around the regions that switch from B to A compartment (Fig 6D). These observations suggest that cell type-specific active chromatin interactions that are involved in transcription of marker gene expression are largely captured by cell type-specific topics, just as cisTopic was able to distinguish cell types from the single-cell ATAC-seq data by detecting cell type-specific accessible regions of chromatin [15].

## Discussion

We have demonstrated that by applying topic modeling to *in silico* mixed sci-Hi-C data, cells can be successfully clustered by their cell type identities, and the learned topics themselves exhibit statistically significant patterns of enrichment relative to chromatin compartments. The decomposition of the sci-Hi-C data into the cell-topic and LP-topic matrices enables visualization of structure that is not otherwise apparent in the data. This decomposition also facilitates investigation of the “meaning” of topics relative to features such as chromatin compartment structure. We envision that topic modeling will be valuable in cases where single cell Hi-C is performed on a complex tissue with an unknown mixture of cell types. In such settings, topic modeling will greatly aid in identifying cell types and discovering latent chromatin topics that reflect the compartment structures of different cell types.

Furthermore, the ability of the topic model to capture cell cycle effects means the effect of cell cycle on 3D chromatin organization can be factored out from Hi-C data. Most bulk Hi-C datasets are performed on cycling cells; thus, the resulting data is a mixture of different 3D genome conformations of cells at different stages of the cell cycle [33]. By collecting single cell Hi-C datasets and applying our model to segregate cells at different stages of the cell cycle, we can potentially remove cell cycle effects from Hi-C data and obtain a more robust picture of the 3D conformation of specific cell types.

Topic modeling offers several important benefits in the context of sci-Hi-C data. The method was specifically developed to operate on sparse data such as sci-Hi-C data. The topics themselves potentially provides clues about which locus pairs are important in driving differences among cell types. In contrast, existing single-cell Hi-C analysis methods HiCRep/MDS and scHiCluster mainly provide an embedding of the data, without offering the topic structure.

In our analysis, we investigated whether topics are associated with cell type-specific locus pair compartment structures. Given the enrichment of contacts made with B-to-A compartment switching regions in cell type-specific topics and that of upregulated cell type-specific genes in the regions that undergo B-to-A compartment transition, we suspect that the cell type-specific topics consist of active chromatin interactions involved in transcription of cell type-specific genes. However, a more complete understanding of this structure may require also investigating finer-scale chromatin properties, such as topologically associating domain structure, or complementary genomic or epigenomic measurements of transcription, factor binding, or histone modifications. Such analysis is challenging because of the necessarily coarse scale (500 kb bin size in this work) of the topic modeling, given the limitations of current single cell Hi-C assays, especially sci-Hi-C. With potential future developments that can yield higher coverage single cell Hi-C data on a large number cells, our topic model has great promise to assist in the study of the dynamics of 3D organization of the genome at finer scale, such as topological domains and loops.

## Supporting information

S1 Fig Most intra-chromosomal contact counts are concentrated at the 5-20Mb range. (A) Percentage of observed intra-chromosomal contacts at different locus pair distances. Barplots showing the number of all possible locus pairs at different values of locus pair distance (B) and resolution of the contact matrices (C).
(TIF)

S2 Fig Selecting the number of topics. (Left) The figure plots the log-likelihood value at the last iteration of training as a function of the number of topics (5–135). (Right) The figure plots the Jensen-Shannon divergence of locus pair normalized topic assignment values between all pairs of topics. Red “X” marks the optimal topic number that has the highest log-likelihood value at 90 topics (left) and Jensen-Shannon divergence value at 30 topics (right).
(TIF)

S3 Fig Topic modeling results show little to no batch effects. (A) Two-dimensional UMAP projection of the cell-topic matrix from Fig 2A is colored by their library identity.
(TIF)

S4 Fig Performance of topic modeling results on downsampled data. (A) Two-dimensional UMAP projection of the cell-topic matrix trained on the downsampled data. (B) Violin plots of silhouette coefficients computed from the UMAP embeddings for each cell type for the downsampled and the original data.
(TIF)

S5 Fig Topic modeling results show slight coverage effects. Two-dimensional UMAP projection of the cell-topic matrix from Fig 2A is colored by coverage.
(TIF)

S6 Fig Comparing topic modeling clustering performance on scHiCluster filtered data. (A) PCA, scHiCluster, and LDA were performed on the scHiCluster filtered data (n = 2258 cells) and the dimensionality reduced data was embedded using UMAP. (B) Violin plots of silhouette coefficients computed from the UMAP embeddings for each cell type.
(TIF)

S7 Fig Visualization of the topic-cell matrix. The resulting topic-cell is normalized by the total number of topic assignments for each topic and visualized. Columns and rows are hierarchically clustered using Euclidean distance with Ward’s clustering algorithm. Colors bars indicate normalized topic assignment values.
(TIF)

S8 Fig UMAP embeddings of topic-cell matrices produced with contact matrices binned at different resolutions. (A) Embeddings of the topic-cell matrices produced at 100kb resolution with topic numbers 5,10, and 25. (B) Embeddings of the topic-cell matrices produced at 250kb, 500kb, and 1Mb resolution.
(TIF)

S9 Fig Comparing topic modeling clustering performance of different sci-Hi-C resolutions. Violin plots of silhouette coefficients computed from the UMAP embeddings in S8 Fig for each cell type, colored by the resolution of the data. Baseline (PCA; 500kb) silhouette coefficients are computed as a reference.
(TIF)

S10 Fig UMAP embeddings of topic-cell matrices produced with different inter locus pair distances. Topic modeling was applied to cell-LP matrices that were produced by varying inter locus pair distance values, and the resulting cell-topic matrices were embedded into UMAP space. Baseline (PCA; 10Mb) embedding is shown as a reference.
(TIF)

S11 Fig Comparing topic modeling clustering performance of different maximum locus pair distances. Violin plots of silhouette coefficients computed from the UMAP embeddings in S10 Fig for each cell type, colored by their maximum locus pair distances.
(TIF)

S12 Fig Comparison of computational performance of LDA at different resolutions. Runtime (A) and maximum CPU memory used (B) on an AMD Opteron 6380, 2.5GHz to train the topic model with 50 topics on matrices binned at 100kb, 250kb, 500kb and 1Mb resolution.
(TIF)

S13 Fig Topic modeling groups cells with a high proportion of mitotic contacts. (A) Two-dimensional UMAP projection of cell-topic matrix from Fig 2A (left), with an unknown cluster of cells from all five cell types highlighted (right). (B) Violin plots of percentages of mitotic (2–12Mb) and short range (< 2Mb) contacts observed in the cell mixture and the rest of the cells. P-values by one sided Wilcoxon tests: *** < 0.001. (C) Plot of percentage of mitotic vs. short range contacts per cell in our datasets, with cells from the cell mixture highlighted in red.
(TIF)

S14 Fig Topic modeling discovers topics specific to oocytes and zygotes. Two-dimensional UMAP projection of topic-cell matrix obtained by applying LDA to Flyamer et al. data, colored by cell types (A) and coverage (B).
(TIF)

S15 Fig Analysis of compartment calls for cell type-specific topic associated locus pairs within cell type. (A) The proportions of GM12878 compartment labels were computed for LPs in topic 24, which is a GM12878 specific topic (bottom), and for LPs in all topics to estimate the expected proportions of GM12878 compartment labels (top). (B) Heatmaps showing the log_2_ ratio of observed over expected proportion of each LP compartment structure in topics specific to each cell type. (C) Stacked bar plots showing the proportion of enriched compartment structure in the cell type-specific topics. Compartment labels were considered enriched in cell type-specific topics if the observed ratio was 25% higher than that of all topics.
(TIF)

S16 Fig IMR90 is the most sparse and least abundant in our datasets. (A) Distribution of log10 contact counts per cell and (B) number of cells for each cell type in our datasets.
(TIF)

S17 Fig Locus pairs in non cell type-specific topics are depleted in compartment switching regions. (A) Barplot showing log_2_ enrichment of locus pairs with compartment switching and stable regions in non cell type-specific topics vs all topics for each cell type. P-values by chi-square test: ** < 0.01, *** < 0.001. (B) Heatmap of log_2_ enrichment of A/B compartment labels in compartment switching regions that are in non cell type-specific vs. all topics.
(TIF)
