# Supplementary figures and images for: Capturing cell type-specific chromatin compartment patterns by applying topic modeling to single-cell Hi-C data
Source: PLoS Comput Biol. 2020 Sep 18;16(9):e1008173. doi: 10.1371/journal.pcbi.1008173 (PMC7526900; doi:10.1371/journal.pcbi.1008173)

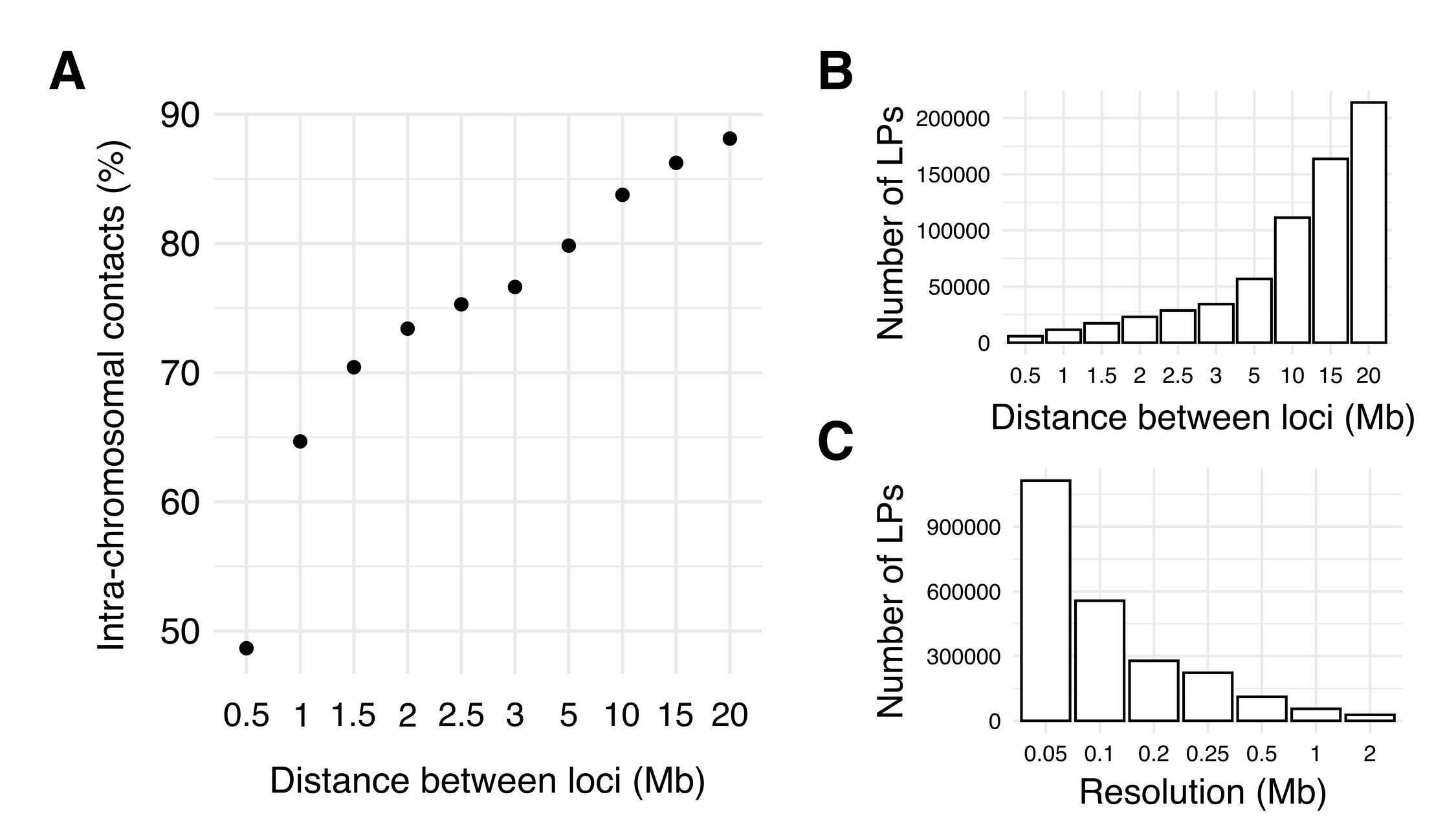

Supplement: S1 Fig — (A) Percentage of observed intra-chromosomal contacts at different locus pair distances. Barplots showing the number of all possible locus pairs at different values of locus pair distance (B) and resolution of the contact matrices (C). (TIF) [file pcbi.1008173.s001.tif]

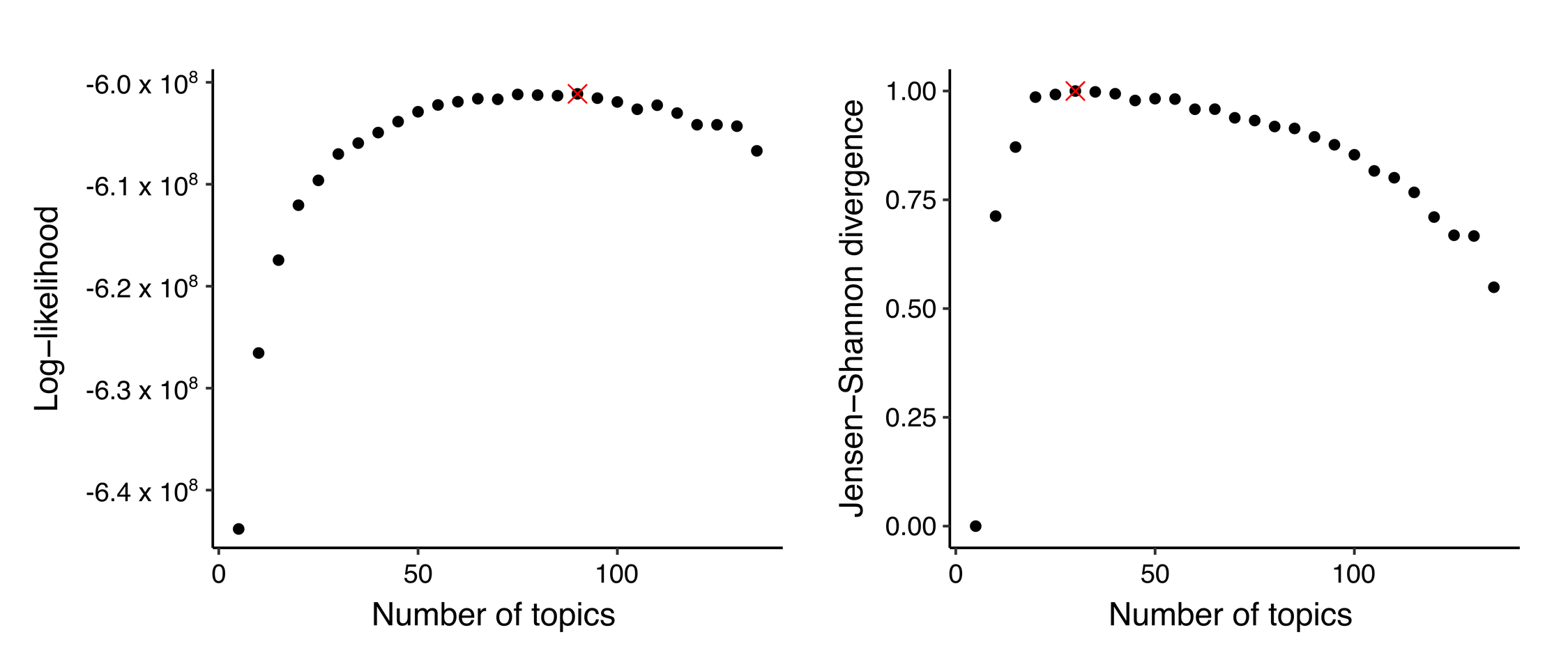

Supplement: S2 Fig — (Left) The figure plots the log-likelihood value at the last iteration of training as a function of the number of topics (5–135). (Right) The figure plots the Jensen-Shannon divergence of locus pair normalized topic assignment values between all pairs of topics. Red “X” marks the optimal topic number that has the highest log-likelihood value at 90 topics (left) and Jensen-Shannon divergence value at 30 topics (right). (TIF) [file pcbi.1008173.s002.tif]

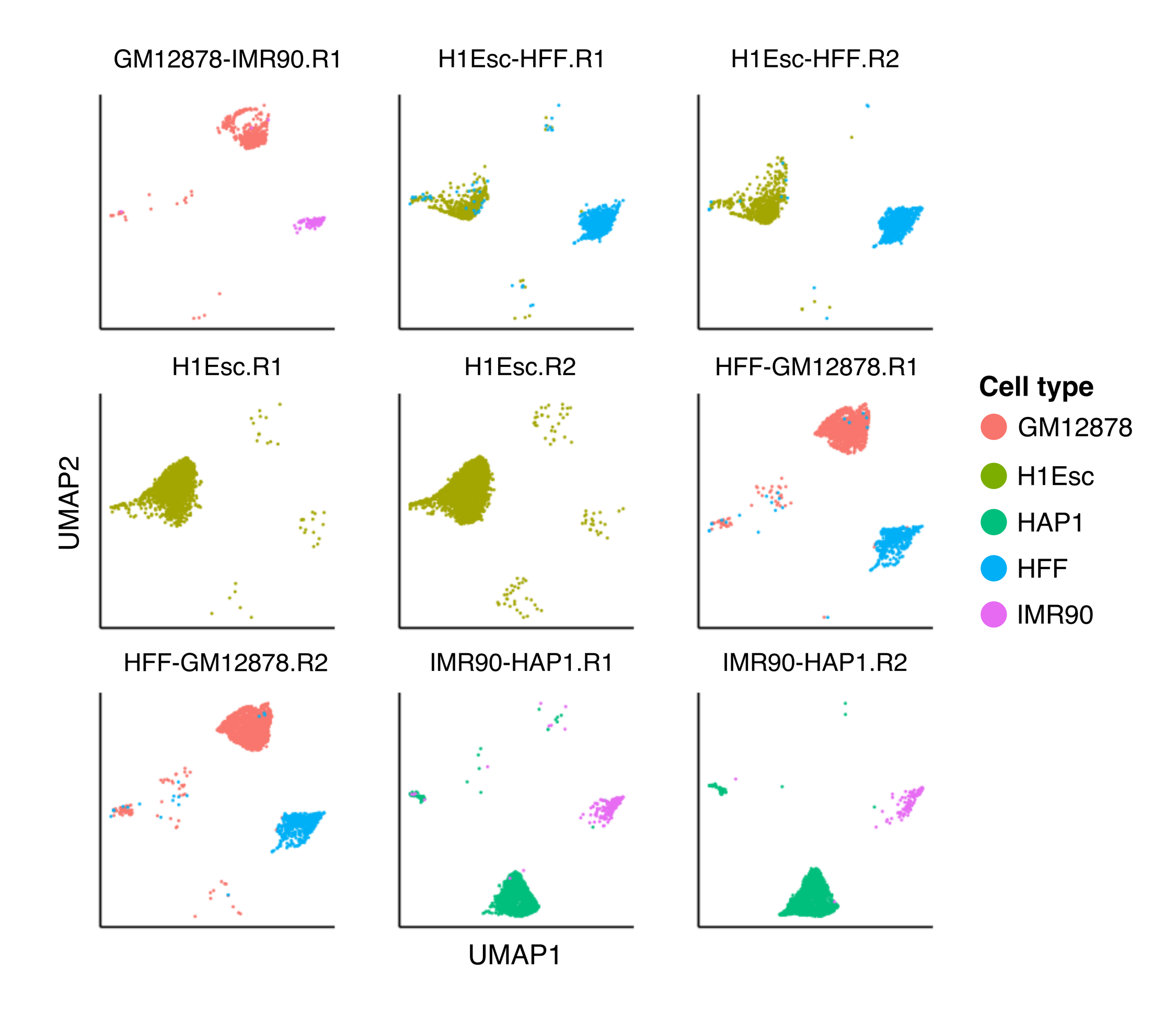

Supplement: S3 Fig — (A) Two-dimensional UMAP projection of the cell-topic matrix from Fig 2A is colored by their library identity. (TIF) [file pcbi.1008173.s003.tif]

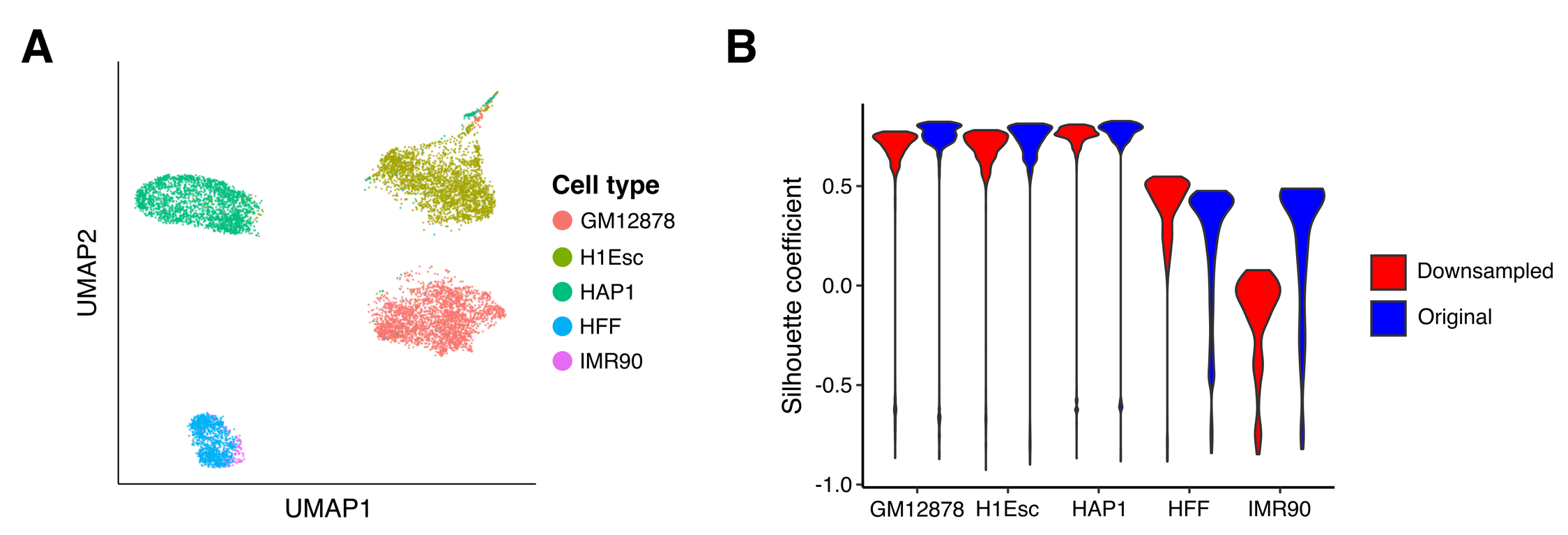

Supplement: S4 Fig — (A) Two-dimensional UMAP projection of the cell-topic matrix trained on the downsampled data. (B) Violin plots of silhouette coefficients computed from the UMAP embeddings for each cell type for the downsampled and the original data. (TIF) [file pcbi.1008173.s004.tif]

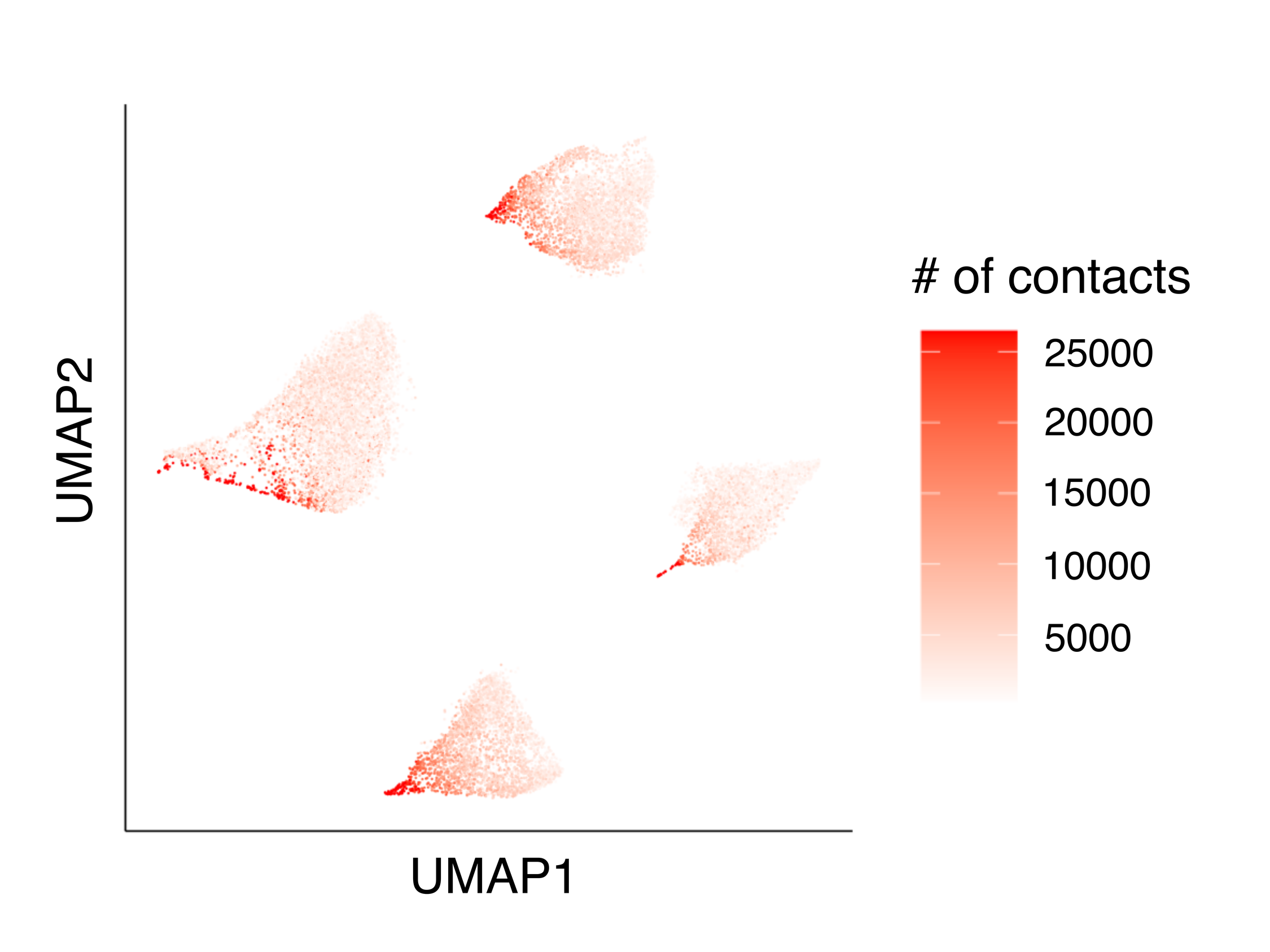

Supplement: S5 Fig — Two-dimensional UMAP projection of the cell-topic matrix from Fig 2A is colored by coverage. (TIF) [file pcbi.1008173.s005.tif]

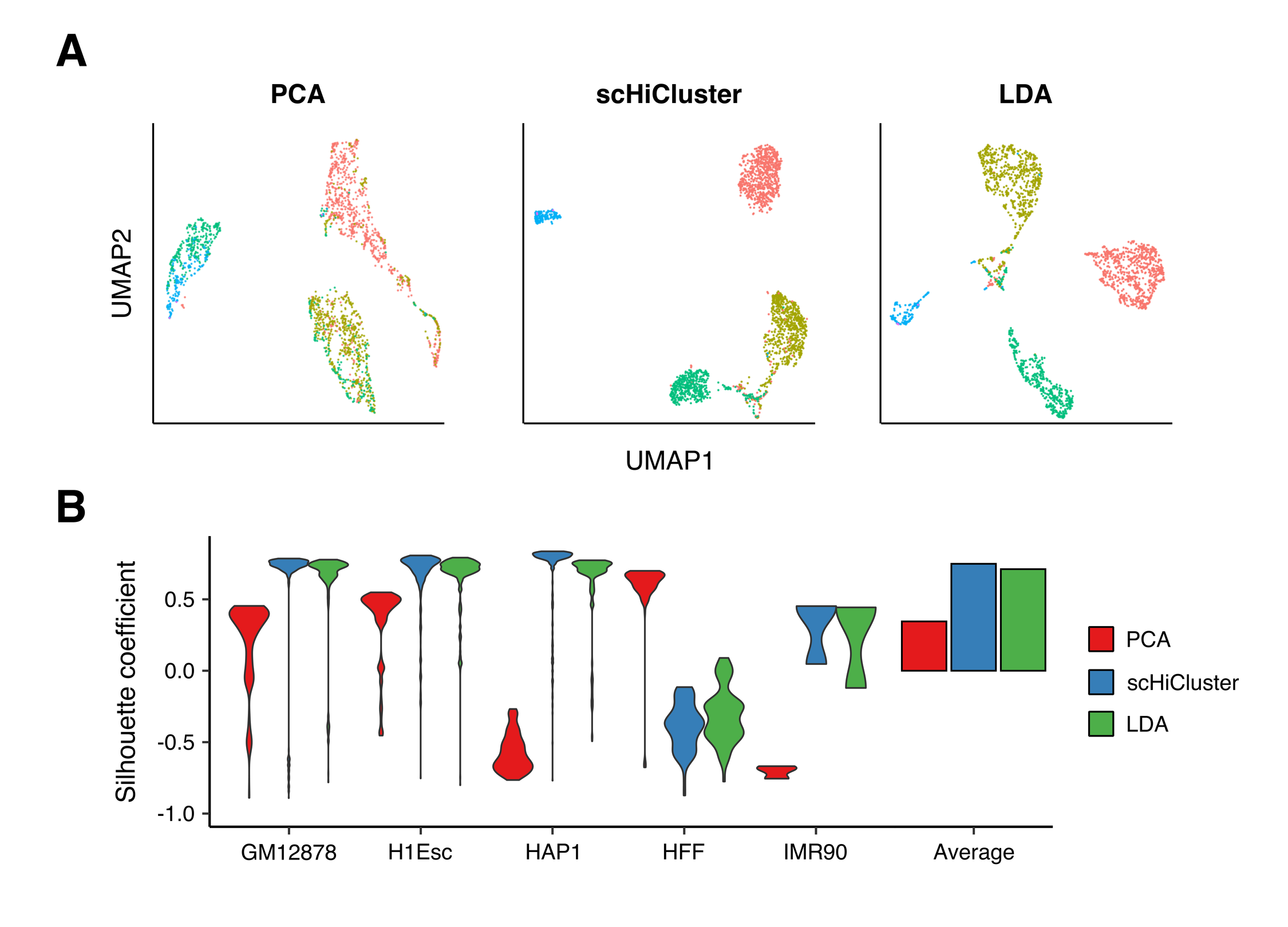

Supplement: S6 Fig — (A) PCA, scHiCluster, and LDA were performed on the scHiCluster filtered data (n = 2258 cells) and the dimensionality reduced data was embedded using UMAP. (B) Violin plots of silhouette coefficients computed from the UMAP embeddings for each cell type. (TIF) [file pcbi.1008173.s006.tif]

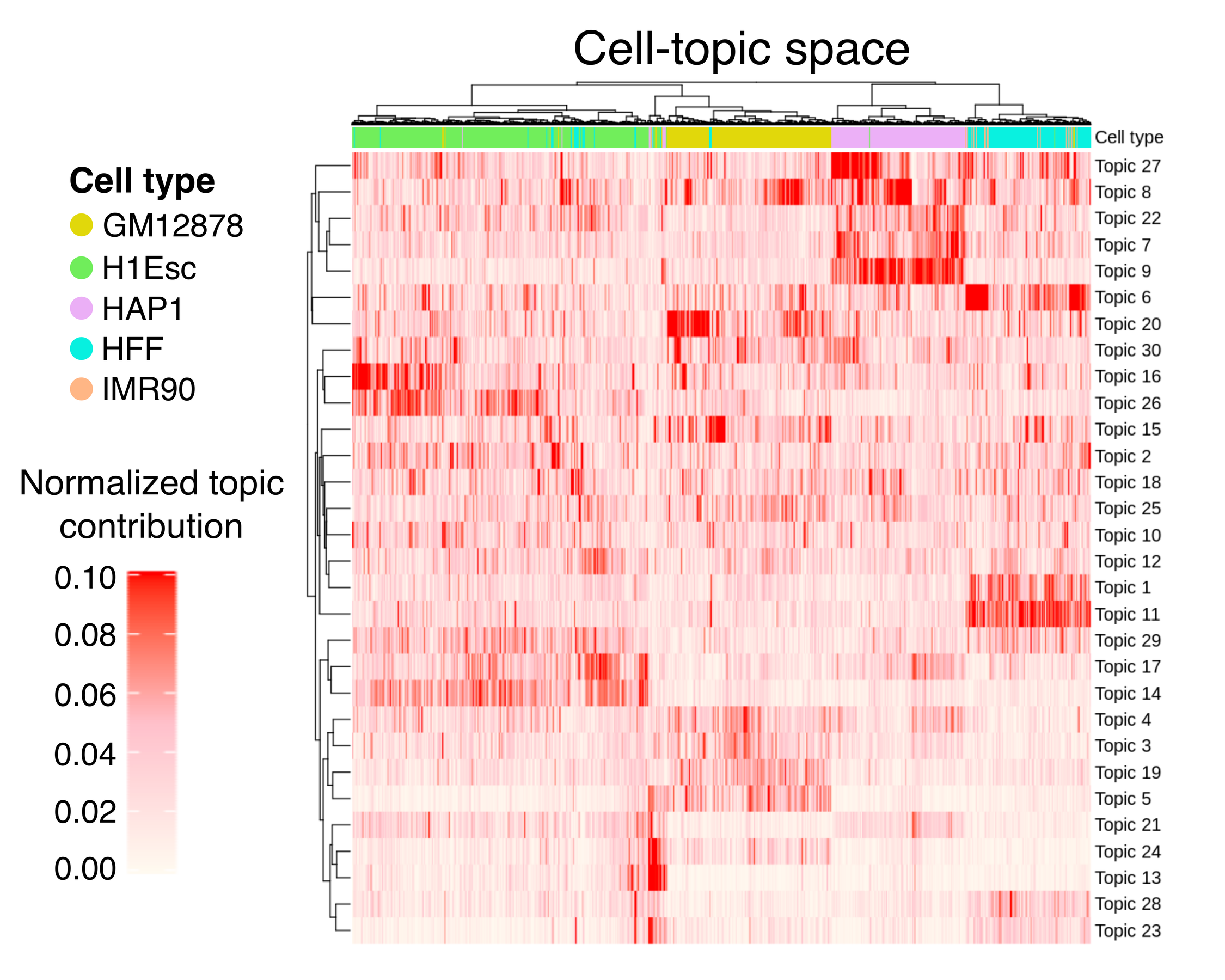

Supplement: S7 Fig — The resulting topic-cell is normalized by the total number of topic assignments for each topic and visualized. Columns and rows are hierarchically clustered using Euclidean distance with Ward’s clustering algorithm. Colors bars indicate normalized topic assignment values. (TIF) [file pcbi.1008173.s007.tif]

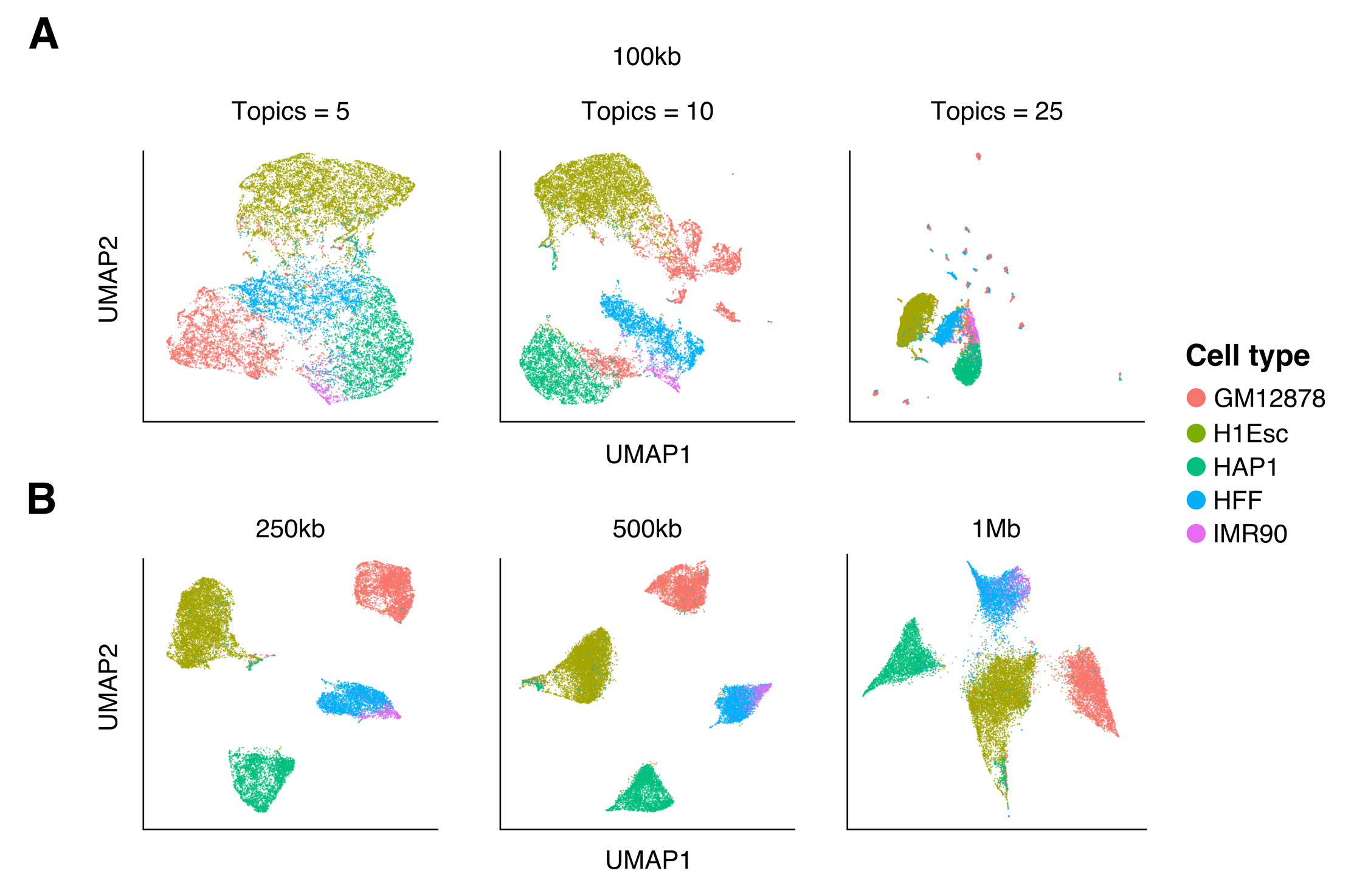

Supplement: S8 Fig — (A) Embeddings of the topic-cell matrices produced at 100kb resolution with topic numbers 5,10, and 25. (B) Embeddings of the topic-cell matrices produced at 250kb, 500kb, and 1Mb resolution. (TIF) [file pcbi.1008173.s008.tif]

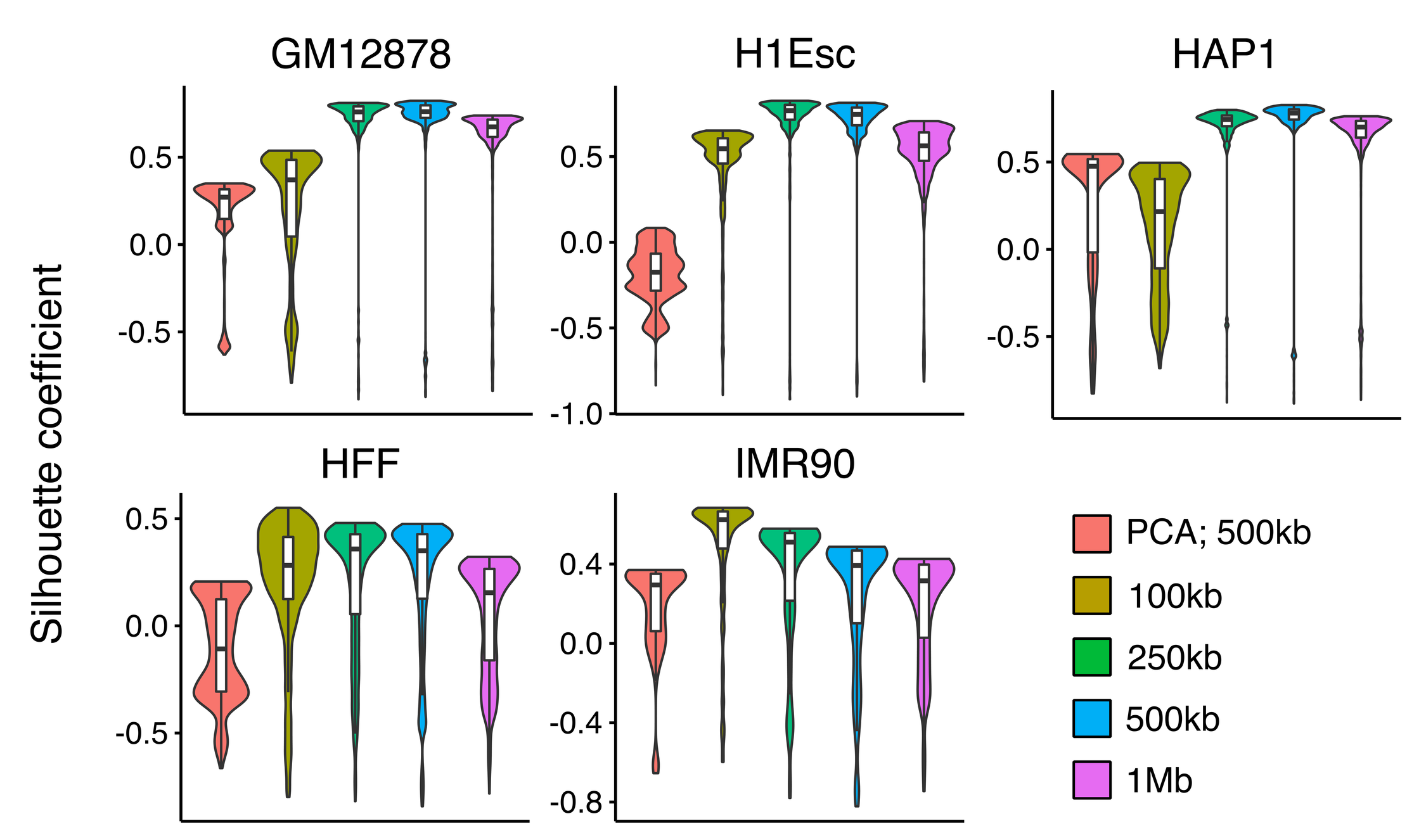

Supplement: S9 Fig — Violin plots of silhouette coefficients computed from the UMAP embeddings in S8 Fig for each cell type, colored by the resolution of the data. Baseline (PCA; 500kb) silhouette coefficients are computed as a reference. (TIF) [file pcbi.1008173.s009.tif]

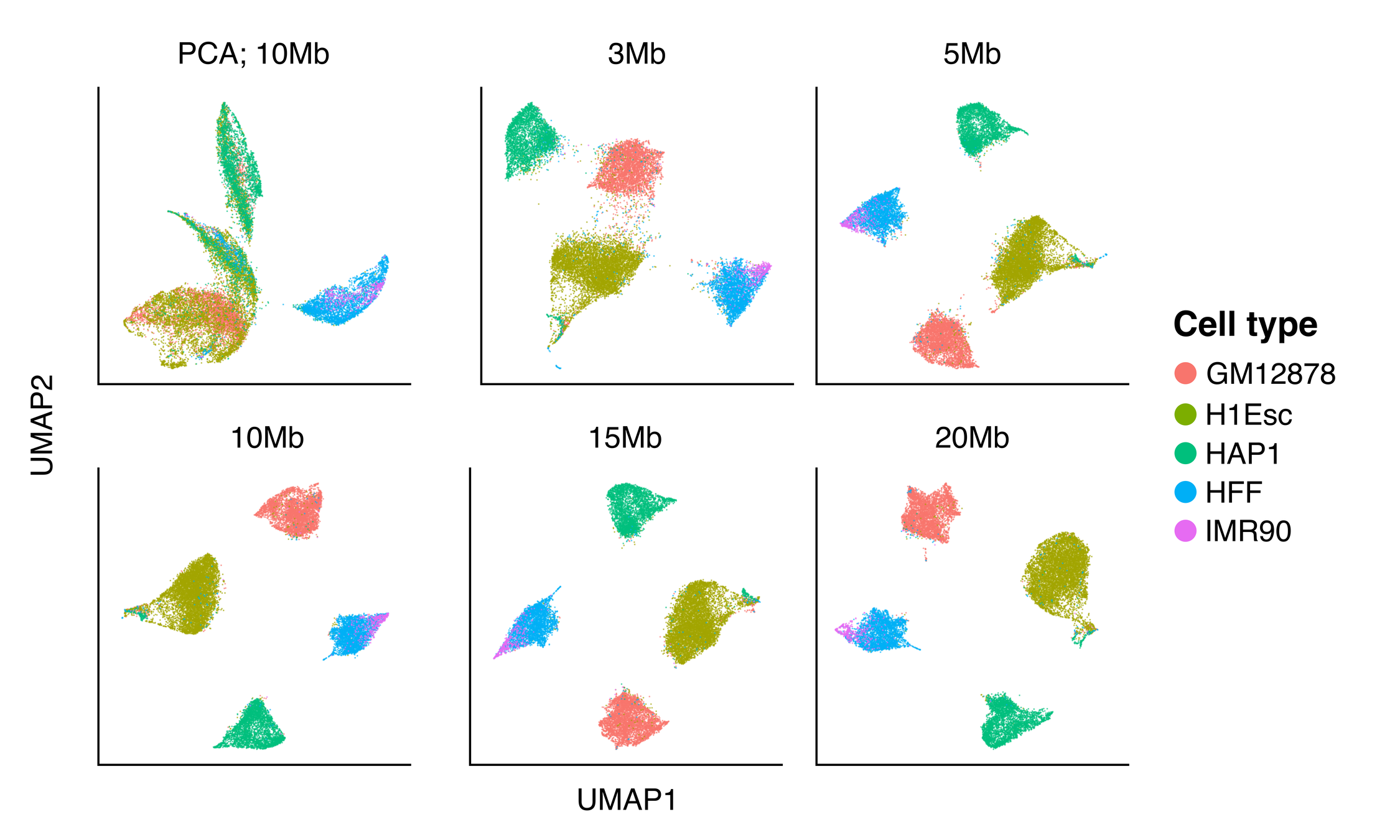

Supplement: S10 Fig — Topic modeling was applied to cell-LP matrices that were produced by varying inter locus pair distance values, and the resulting cell-topic matrices were embedded into UMAP space. Baseline (PCA; 10Mb) embedding is shown as a reference. (TIF) [file pcbi.1008173.s010.tif]

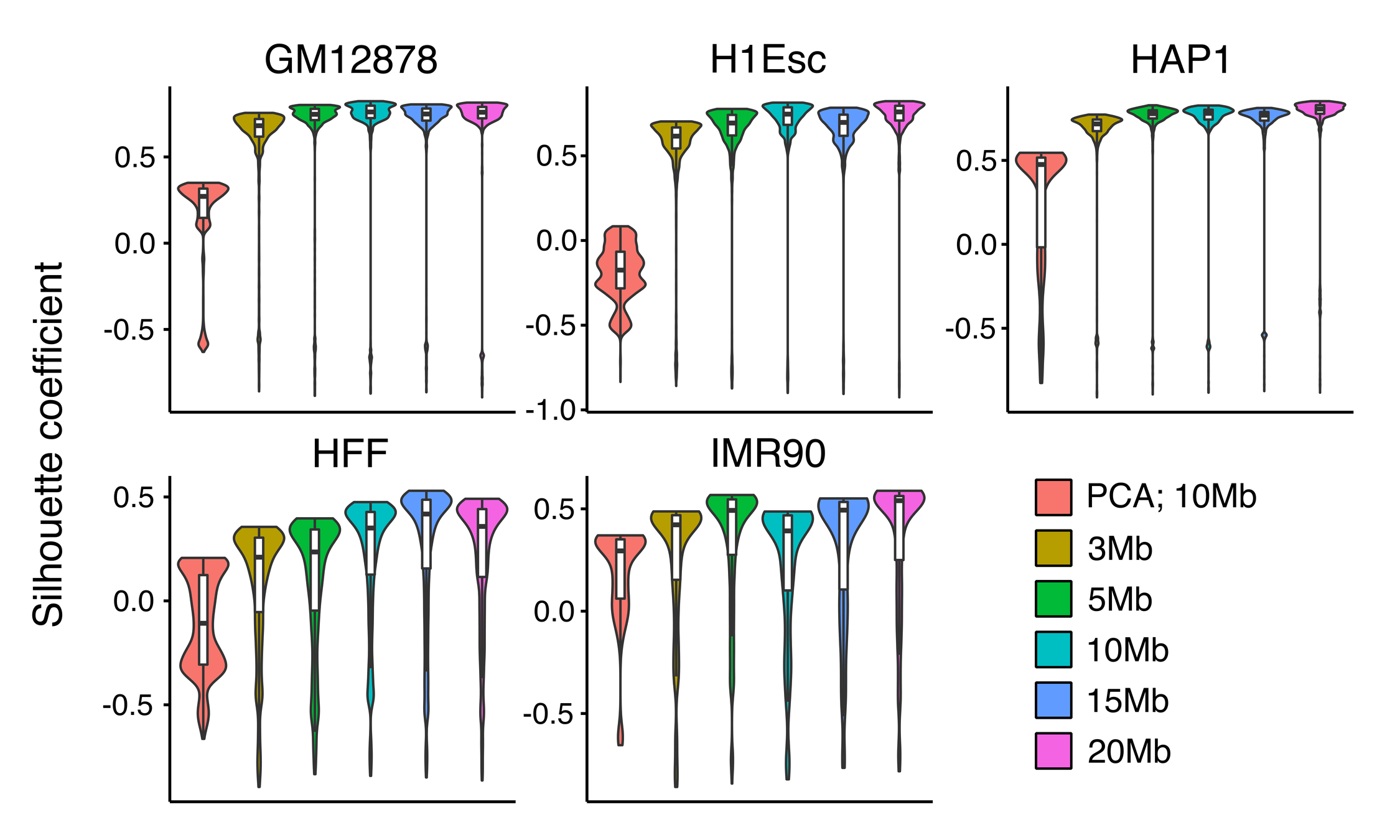

Supplement: S11 Fig — Violin plots of silhouette coefficients computed from the UMAP embeddings in S10 Fig for each cell type, colored by their maximum locus pair distances. (TIF) [file pcbi.1008173.s011.tif]

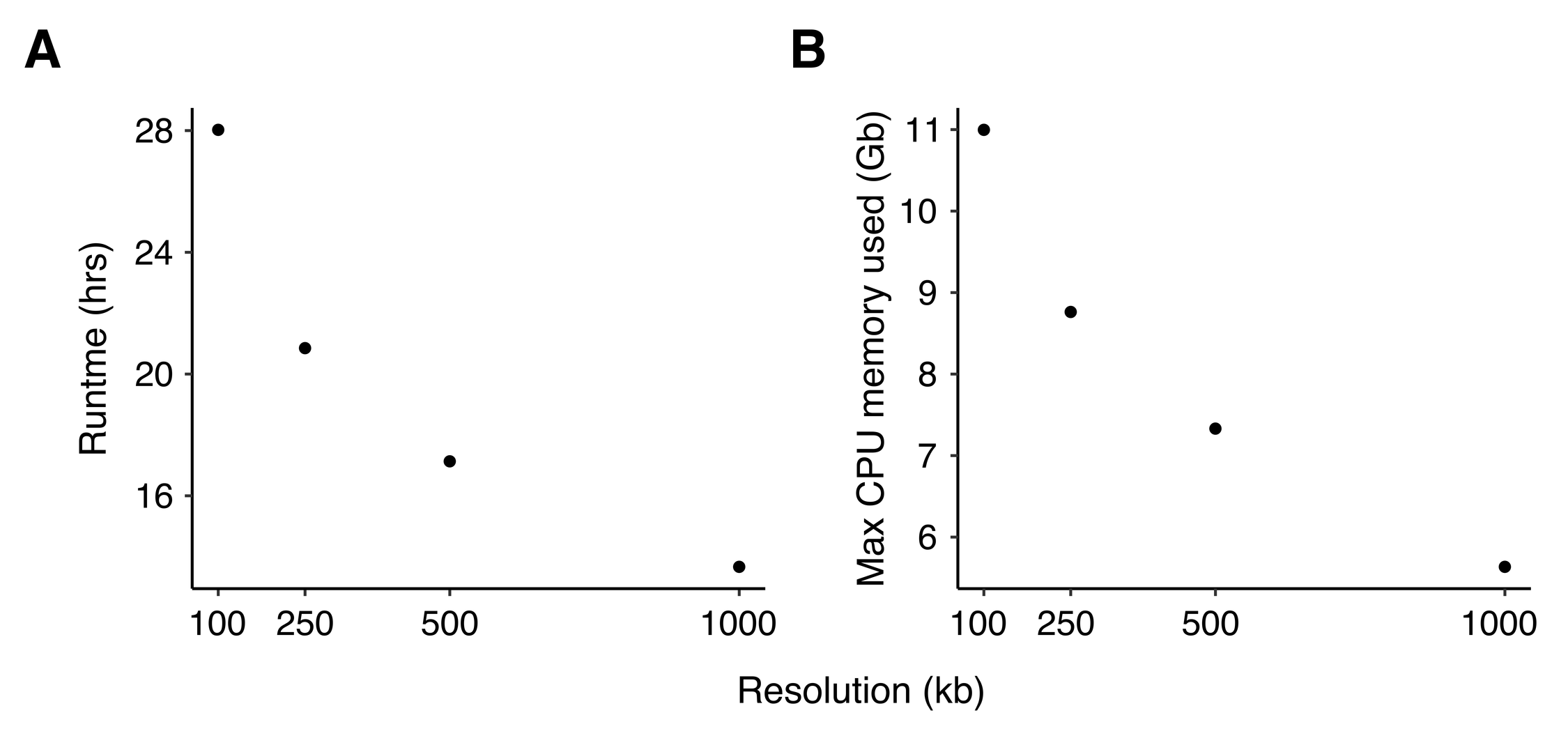

Supplement: S12 Fig — Runtime (A) and maximum CPU memory used (B) on an AMD Opteron 6380, 2.5GHz to train the topic model with 50 topics on matrices binned at 100kb, 250kb, 500kb and 1Mb resolution. (TIF) [file pcbi.1008173.s012.tif]

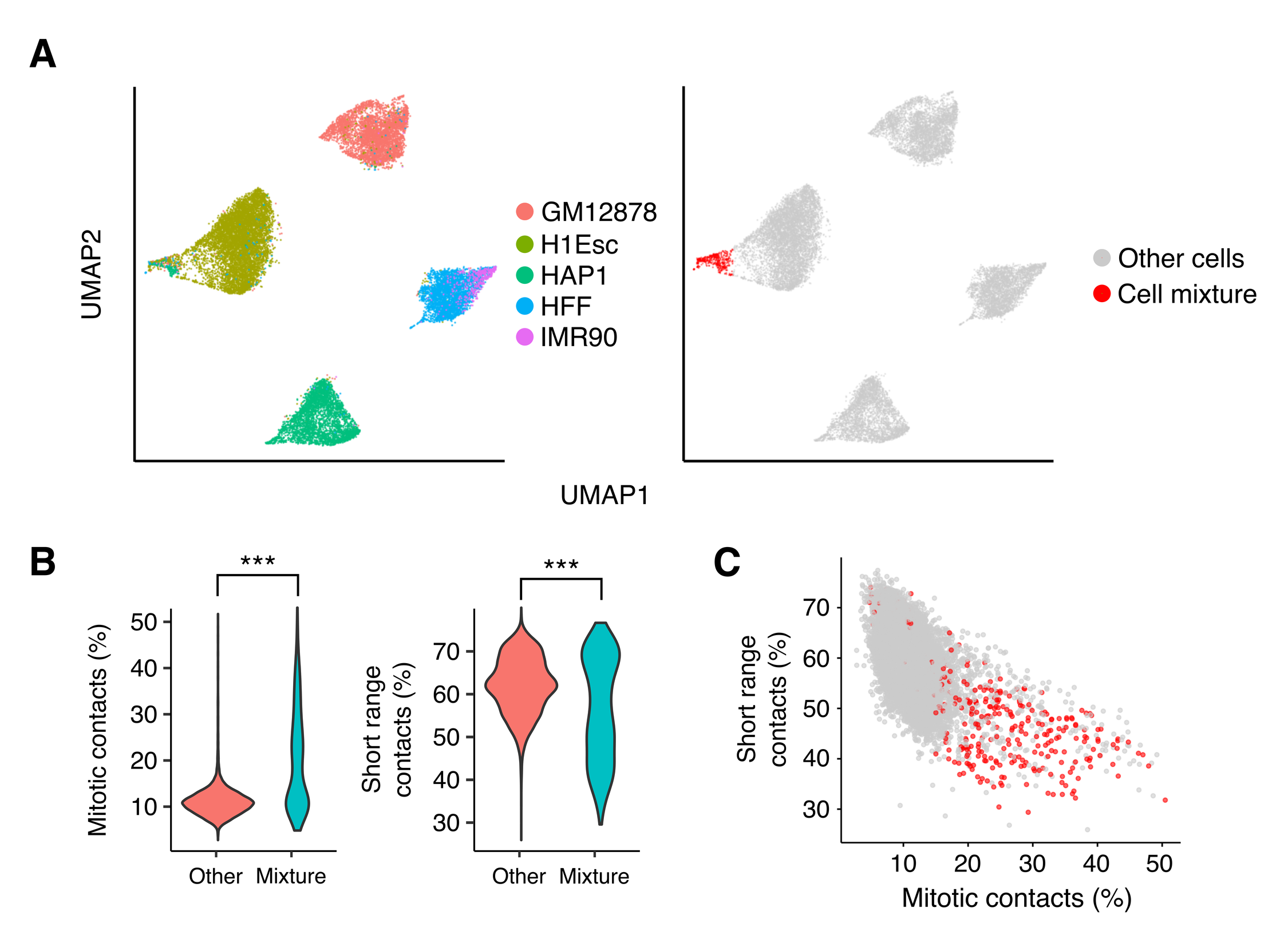

Supplement: S13 Fig — (A) Two-dimensional UMAP projection of cell-topic matrix from Fig 2A (left), with an unknown cluster of cells from all five cell types highlighted (right). (B) Violin plots of percentages of mitotic (2–12Mb) and short range (< 2Mb) contacts observed in the cell mixture and the rest of the cells. P-values by one sided Wilcoxon tests: *** < 0.001. (C) Plot of percentage of mitotic vs. short range contacts per cell in our datasets, with cells from the cell mixture highlighted in red. (TIF) [file pcbi.1008173.s013.tif]

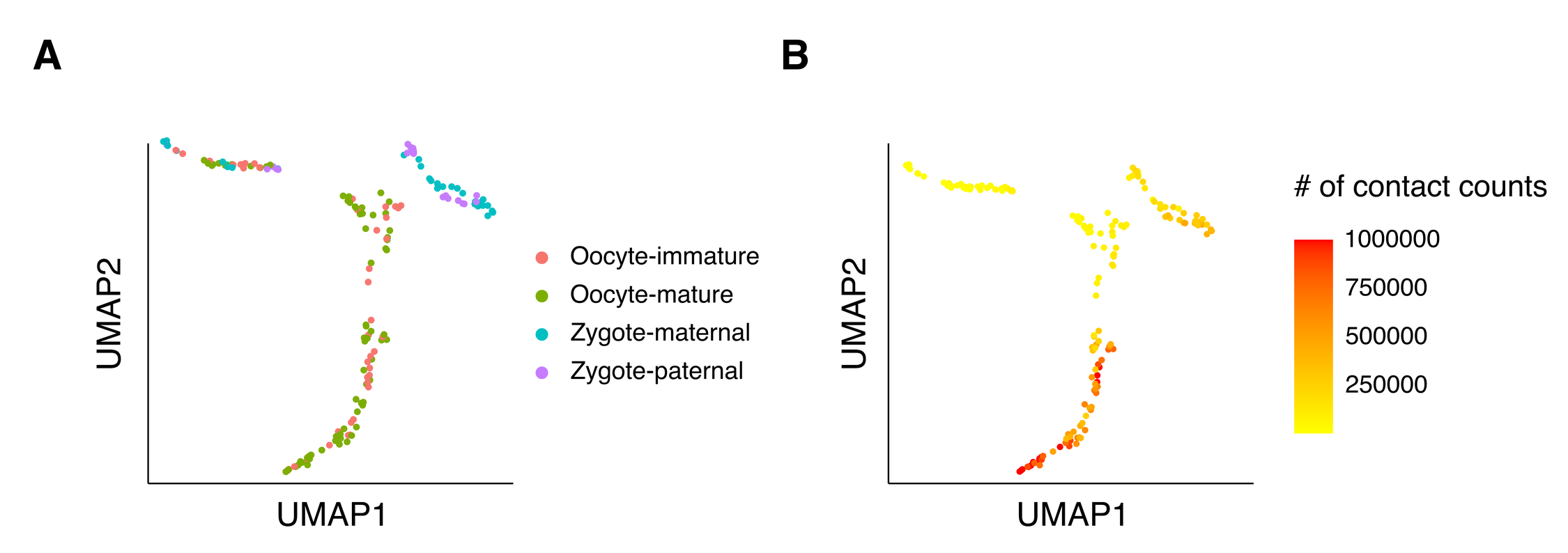

Supplement: S14 Fig — Two-dimensional UMAP projection of topic-cell matrix obtained by applying LDA to Flyamer et al. data, colored by cell types (A) and coverage (B). (TIF) [file pcbi.1008173.s014.tif]

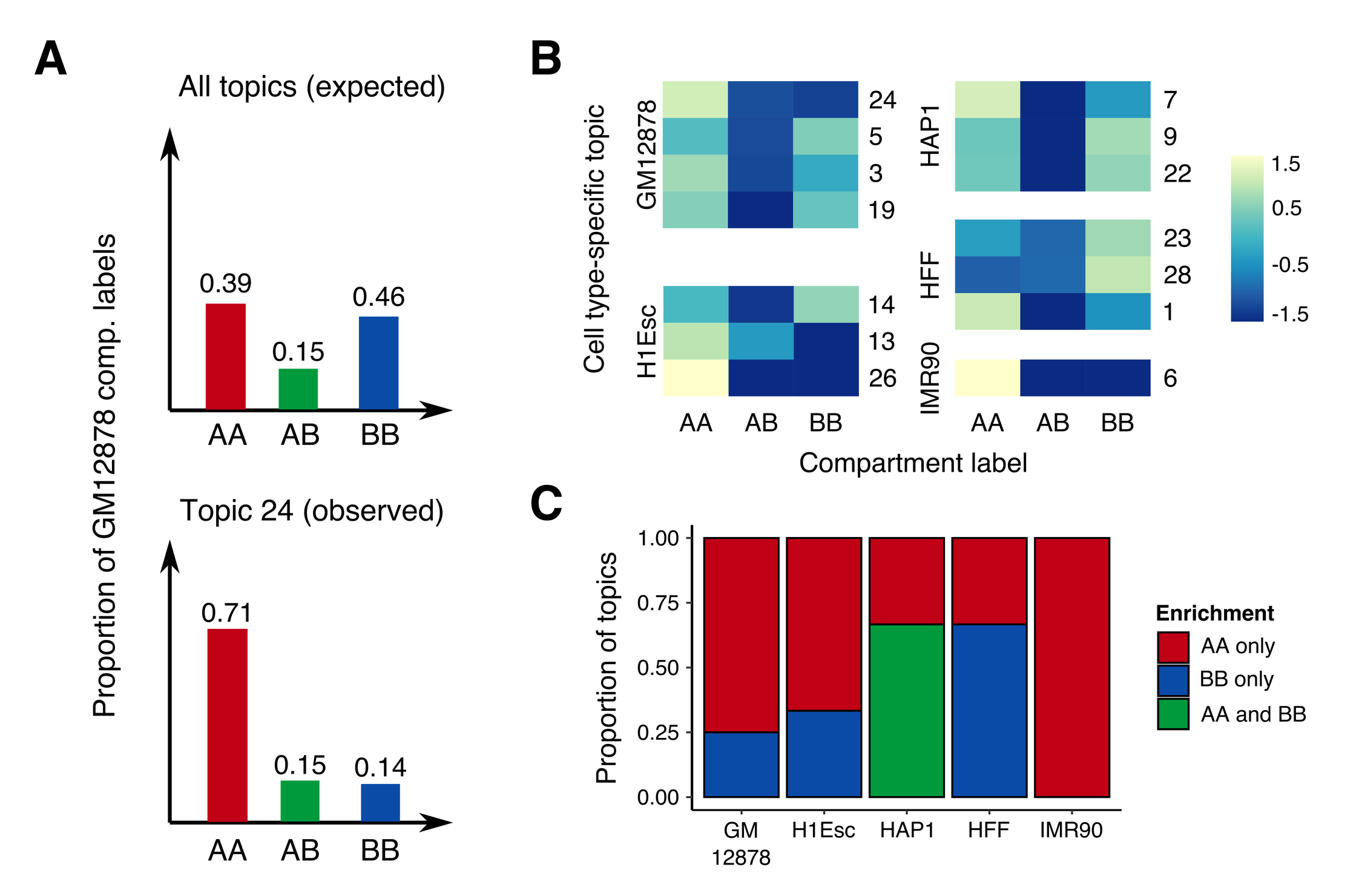

Supplement: S15 Fig — (A) The proportions of GM12878 compartment labels were computed for LPs in topic 24, which is a GM12878 specific topic (bottom), and for LPs in all topics to estimate the expected proportions of GM12878 compartment labels (top). (B) Heatmaps showing the log2 ratio of observed over expected proportion of each LP compartment structure in topics specific to each cell type. (C) Stacked bar plots showing the proportion of enriched compartment structure in the cell type-specific topics. Compartment labels were considered enriched in cell type-specific topics if the observed ratio was 25% higher than that of all topics. (TIF) [file pcbi.1008173.s015.tif]

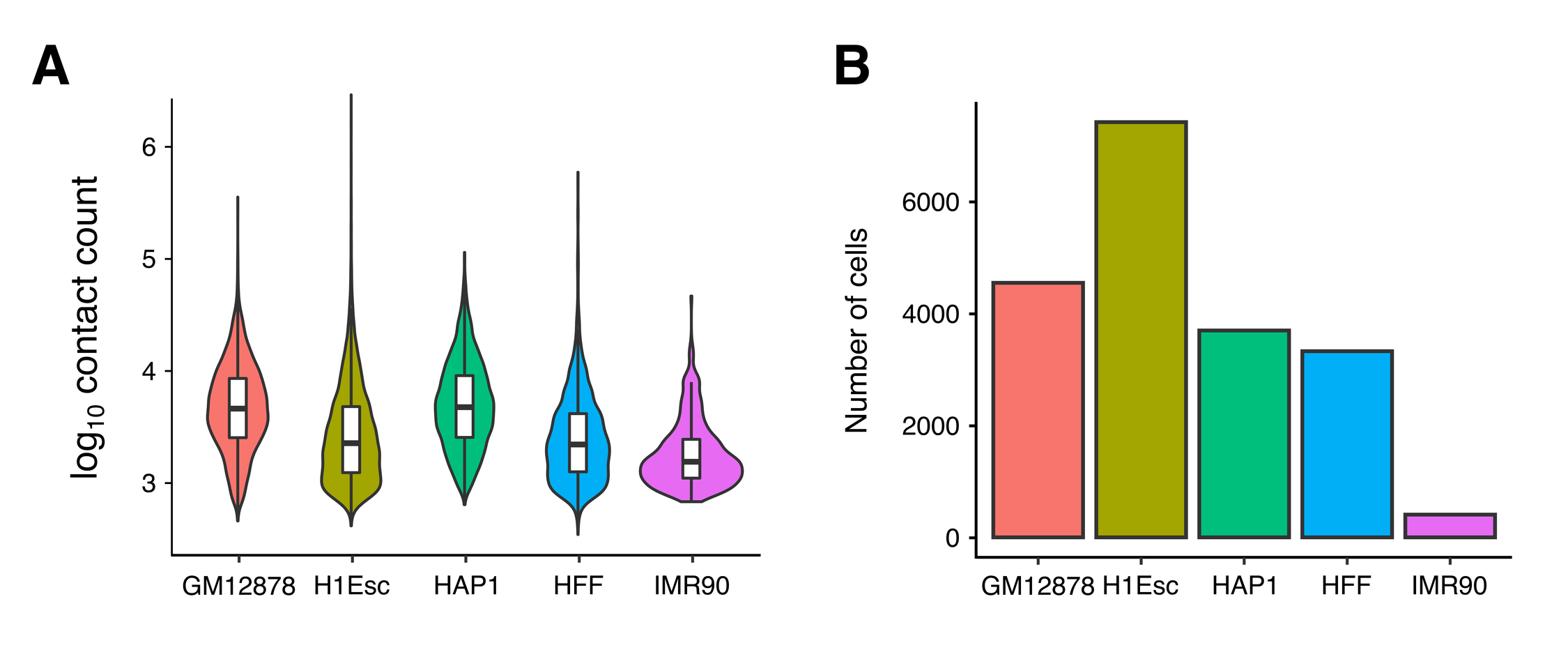

Supplement: S16 Fig — (A) Distribution of log10 contact counts per cell and (B) number of cells for each cell type in our datasets. (TIF) [file pcbi.1008173.s016.tif]

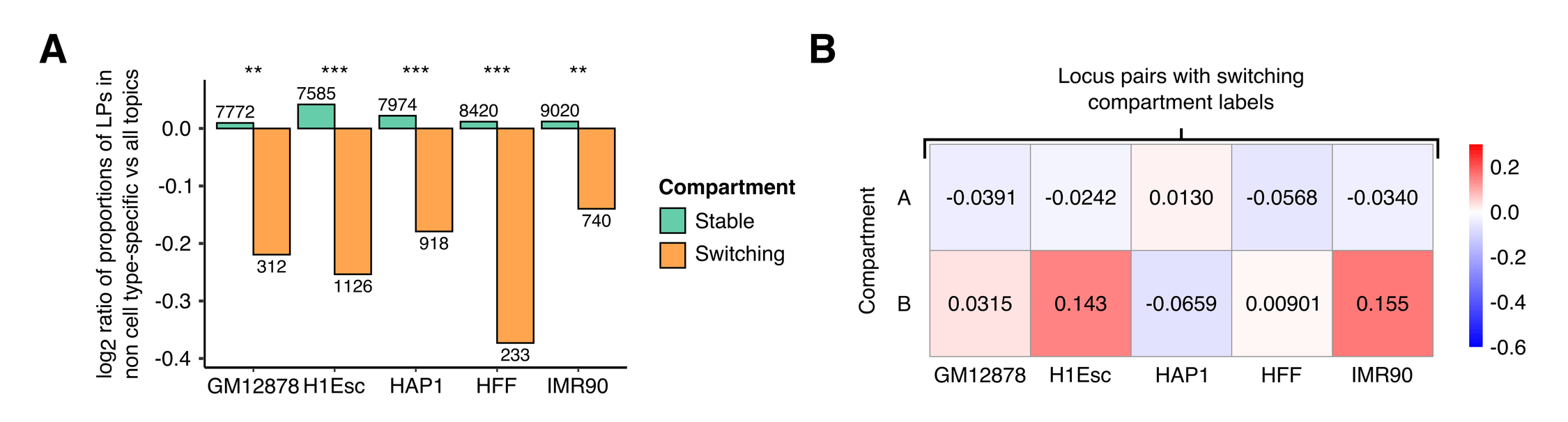

Supplement: S17 Fig — (A) Barplot showing log2 enrichment of locus pairs with compartment switching and stable regions in non cell type-specific topics vs all topics for each cell type. P-values by chi-square test: ** < 0.01, *** < 0.001. (B) Heatmap of log2 enrichment of A/B compartment labels in compartment switching regions that are in non cell type-specific vs. all topics. (TIF) [file pcbi.1008173.s017.tif]
